# Stabilities of the Divalent Metal Ion Complexes of a Short-Chain Polyphosphate Anion and Its Imino Derivative

**DOI:** 10.1007/s10953-013-0099-2

**Published:** 2013-11-07

**Authors:** Hideshi Maki, Masahiko Tsujito, Makoto Sakurai, Tetsuji Yamada, Hiroyuki Nariai, Minoru Mizuhata

**Affiliations:** 1 Department of Chemical Science and Engineering, Graduate School of Engineering, Kobe University, 1-1 Rokkodai-cho, Nada, Kobe, 657-8501 Japan; 2 Department of Applied Chemistry, College of Engineering, Chubu University, 1200 Matsumoto-cho, Kasugai, Aichi 487-8501 Japan

**Keywords:** Imido phosphate, Entropy, Enthalpy, Schwarzenbach, Potentiometric titration, Multidentate complex, Irving–Williams order

## Abstract

**Abstract:**

The stability constants of ML-type complexes of the two linear triphosphate ligand anion analogues triphosphate (${\text{P}}_{ 3} {\text{O}}_{10}^{5 - }$) and diimidotriphosphate (${\text{P}}_{ 3} {\text{O}}_{ 8} ( {\text{NH}})_{2}^{5 - }$) were investigated thermodynamically using potentiometric titrations according to Schwarzenbach’s procedure. The stability constants of the ML-type complexes of different divalent metal ions with ${\text{P}}_{ 3} {\text{O}}_{ 8} ( {\text{NH}})_{2}^{5 - }$ are larger than those of the corresponding complexes with ${\text{P}}_{ 3} {\text{O}}_{10}^{5 - }$ because of the greater basicity of the imino group. The order of the stability constants for the ML-type complexes follows the Irving–Williams order, indicating that only non-bridging oxygen atoms are coordinated directly to the different metal ions in both ligands, and that the imino groups cannot participate in coordination to the metal ions. In the complexation reactions of the Ca^2+^, Sr^2+^, Ba^2+^–${\text{P}}_{ 3} {\text{O}}_{10}^{5 - }$ and Cu^2+^, Zn^2+^, Ni^2+^–${\text{P}}_{ 3} {\text{O}}_{ 8} ( {\text{NH}})_{2}^{5 - }$ systems, each metal ion forms an enthalpically stable complex, and there was no suggestion of a conspicuous entropic effect based on the chelate effect. Monodentate complexes that are strongly coordinated with the ligands were therefore formed, whereas entropically stable bidentate complexes were formed in the complexation reactions of the Cu^2+^, Zn^2+^, Ni^2+^–${\text{P}}_{ 3} {\text{O}}_{10}^{5 - }$ and Ca^2+^, Ba^2+^, Sr^2+^–${\text{P}}_{ 3} {\text{O}}_{ 8} ( {\text{NH}})_{2}^{5 - }$ systems. According to the HSAB concept, hard metal cations such as Ca^2+^, Ba^2+^ and Sr^2+^ should bind to the harder oxygen atoms rather than the softer nitrogen atoms of the imidopolyphosphate anions, preventing direct coordination to the imino nitrogen atom.

**Graphical Abstract:**

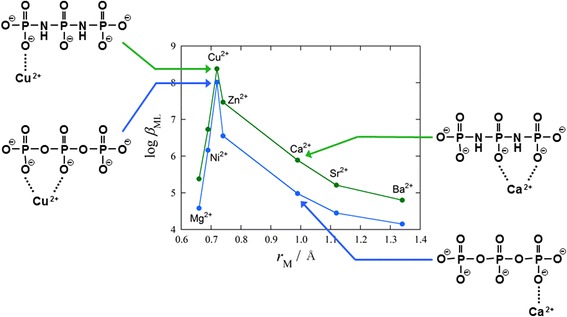

**Electronic supplementary material:**

The online version of this article (doi:10.1007/s10953-013-0099-2) contains supplementary material, which is available to authorized users.

## Introduction

Imidophosphates are phosphorus and nitrogen containing compounds that have been used as chemical fertilizers for some time. In recent years, these compounds have also been used as flame retardants in organic materials, including a variety of plastics and painting materials [1–3]. The chemical stabilities of imidophosphates, however, can be poor under certain conditions. For example, these materials are more readily hydrolyzed than halogen compound-based flame retardants. For this reason, imidophosphates have not been studied to any significant extent.

In recent years, however, numerous reports have appeared in the literature indicating that the addition of specific metal ions to imidophosphates can greatly improve their chemical stability, and this has resulted in extensive research in this area [4–10]. The acid dissociation behaviors and complex formation equilibria of these imidophosphates are believed to be closely related to improvements in their stability properties. Detailed mechanistic studies of these materials have not yet been conducted, however, the complexation behaviors of linear polyphosphate anions in aqueous solutions (i.e., the complex species and their distributions) are influenced by changes in the effective charges of the anions, which are dependent on the number of protons binding to the anions. It would therefore be useful to study the complexation equilibria between the linear polyphosphate anions and a variety of different metal ions to find ways to improve the hydrolysis resistance of imidophosphate compounds.

**Fig. 1:**
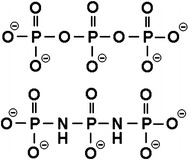
Structures of ${\text{P}}_{ 3} {\text{O}}_{(10 - n)} ({\text{NH}})_{n}^{5 - }$ (*n* = 0, 2) anions. *Upper plot* ${\text{P}}_{ 3} {\text{O}}_{10}^{5 - }$, *lower plot* ${\text{P}}_{ 3} {\text{O}}_{ 8} ( {\text{NH}})_{2}^{5 - }$

The triphosphate anion, ${\text{P}}_{ 3} {\text{O}}_{10}^{5 - }$, and the diimidotriphosphate anion, ${\text{P}}_{ 3} {\text{O}}_{ 8} ( {\text{NH}})_{2}^{5 - }$, investigated in the current study are short-chain polyphosphate anions containing three phosphorus atoms that are connected to each other through oxygen atoms or imino groups, respectively (Fig. 1). The basicity of P–NH–P is greater than that of the P–O–P [11–20], and it was envisaged that the stabilities of the different metal ion complexes of the ${\text{P}}_{ 3} {\text{O}}_{ 8} ( {\text{NH}})_{2}^{5 - }$ anion would be greater than those of the corresponding ${\text{P}}_{ 3} {\text{O}}_{10}^{5 - }$ anion. In our previous work [21–24], we showed that the affinities of a range of different *cyclo*-μ-imidotriphosphate anions to protons and various divalent metal ions increased in a linear manner as the number of imino groups in the anions increased. Furthermore, the complexation reaction of the Cu^2+^– $cyclo{\text{-P}}_{3} {\text{O}}_{6} ({\text{NH}})_{3}^{3 - }$ system is particularly entropically favorable, and the *cyclo*-P_3_O_6_(NH)_3_ ligand was shown to form an intramolecular bidentate complex with a Cu^2+^ ion as a consequence of the stereochemical adjustment of the ligand molecule to the metal ion. The stabilization of the complex resulting from this stereochemical adjustment was suggested as the basis for the complexation equilibria between the cyclic imidopolyphosphates and the different metal ions.

In the current study, stability constants of ML-type complexes of the ${\text{P}}_{ 3} {\text{O}}_{10}^{5 - }$ and ${\text{P}}_{ 3} {\text{O}}_{ 8} ( {\text{NH}})_{2}^{5 - }$ anions with a variety of different divalent metal ions were determined using potentiometric titrations according to Schwarzenbach’s procedure. Furthermore, the enthalpy and the entropy changes that occurred during the ML-type complexation reactions of these anions (i.e., Δ*H*° and Δ*S*°) were determined from the temperature dependence of the complex stability constants, and the coordination structures of the complexes resulting from these anions were estimated. These accurate thermodynamic investigations of the complexation equilibria of the linear polyphosphate anions should provide useful information to enhance our understanding of the influence of different metal ions on the hydrolysis behaviors of imidopolyphosphates.

## Experimental Section

### Chemicals Used

Pentasodium triphosphate hexahydrate, Na_5_P_3_O_10_·6H_2_O, was synthesized and purified according to the literature [25, 26]. *Elemental analysis* Found: Na, 24.52; P, 19.58; O, 33.95; H_2_O, 21.95; calculated for Na_5_P_3_O_10_·6H_2_O: Na, 24.15; P, 19.52; O, 33.62; H_2_O, 22.71 %. The purity was obtained by HPLC and ^31^P NMR measurements, and was found to be over 98 %. Analytical grade of NaNO_3_ (Merck Co. Ltd., 99 %) was purchased from Merck Co. Ltd. (Darmstadt, Germany) and used without further purification. A standard NaNO_3_ stock solution was prepared at about 3.0 mol·L^−1^ with distilled water to be used upon dilution as supporting electrolyte. A portion of the stock solution was dried perfectly at 110 °C at least 5 days, and thus the molality of the stock solution were determined gravimetrically. A HNO_3_ stock solution was prepared at about 0.25 mol·L^−1^ from analytical grade HNO_3_ (Wako Pure Chemical Industries, Ltd., 99 %) with distilled water, and was standardized by a titration with KHCO_3_ [27]. A carbonate-free alkaline stock solution was prepared at about 1.0 mol·L^−1^ by dilution of a plastic ampoule of CO_2_-free NaOH aqueous solution (Merck Co. Ltd., No.109959, 99 %) with CO_2_-free water that had been boiled for at least 15 min under N_2_ atmosphere. The stock solution was checked periodically by Gran’s procedure [28], and the carbonate was under 0.5 % of the NaOH present. Standard stock solutions of M(NO_3_)_2_ [M: Mg, Ca, Sr, Ba, Ni, Cu, and Zn (Wako Pure Chemical Industries, Ltd., 98 %)] were prepared at about 0.2 mol·L^−1^ from their respective reagents with distilled water. The molalities of these stock solutions were determined by complexometric titration with EDTA using murexide (M: Ni and Cu), Eriochrome Black T (M: Mg, Sr, Ba, and Zn), and NN diluted with K_2_SO_4_ (M: Ca) as indicator, respectively. Other reagents used in this work were analytical grade of ordinary commercial products (over 98 %).

### Preparation of Na_5_P_3_O_8_(NH)_2_·6H_2_O

Pentasodium di-imidotriphosphate hexahydrate, Na_5_P_3_O_8_(NH)_2_·6H_2_O, was synthesized by an improved hydrolysis method of trisodium *cyclo*-tri-μ-imidotriphosphate tetrahydrate, Na_3_P_3_O_6_(NH)_3_·4H_2_O, as has been reported [29]. Na_3_P_3_O_6_(NH)_3_·4H_2_O was synthesized according to the literature [30]. A 20 g sample of Na_3_P_3_O_6_(NH)_3_·4H_2_O was dissolved in 300 mL of 0.35 mol·L^−1^ acetic acid in a three-necked round-bottom flask, and hydrolyzed at 60 °C for 16 h with stirring. After cooling to room temperature, the reaction product was precipitated by addition of 300 mL of ethanol. The precipitate was collected by suction filtration, washed with 50 mL of ethanol, and dissolved in 300 mL of water. A 130 g sample of NaOH pellets was added gradually to the aqueous solution, and the solution was reacted at 70 °C for 3 h with stirring. The solution temperature should not be over 75 °C. After cooling to room temperature, the reaction product was collected by suction filtration, washed with 25 mL of ethanol and then 50 mL of acetone. This raw Na_5_P_3_O_8_(NH)_2_·6H_2_O was dissolved in 300 mL of 0.10 mol·L^−1^ NaOH aqueous solution, and 25 mL of ethanol was added to the solution. After stirring for several minutes, a white precipitate was collected by suction filtration, washed with 25 mL of ethanol and then with 50 mL of acetone. Acetone is more suitable for the final washing than alcohol for the preparation of various inorganic phosphates, since it is hard for acetone to form hydrogen bonds with the inorganic phosphates.

The precipitate was vacuum dried for 1 day, and 3.3 g of pure Na_5_P_3_O_8_(NH)_2_·6H_2_O was prepared. The total yield was 13 %. *Elemental analysis* Found: Na, 24.77; P, 19.86; O, 26.87; N, 5.85; H_2_O, 22.11; calculated for Na_5_P_3_O_8_(NH)_2_·6H_2_O: Na, 24.25; P, 19.60; O, 27.00; N, 5.91; H_2_O, 22.80 %. The purities of Na_5_P_3_O_10_·6H_2_O and Na_5_P_3_O_8_(NH)_2_·6H_2_O were obtained by HPLC and ^31^P NMR measurements, and were found to be over 98 %. The phosphorus concentration in these ligand stock solutions were determined colorimetrically with a Mo(V)–Mo(VI) reagent [31].

### Potentiometric Titrations for the Determination of the Stepwise Protonation Constants

Stepwise protonation constants of the ${\text{P}}_{ 3} {\text{O}}_{10}^{5 - }$ and ${\text{P}}_{ 3} {\text{O}}_{ 8} ( {\text{NH}})_{2}^{5 - }$ anions were determined by potentiometric titration. All titration procedures were carried out automatically at 5.0, 15.0, 25.0, 30.0, or 35.0 (±0.5) °C under a N_2_ atmosphere, with the use of a personal computer. A potentiometer (Orion 720A Ionalyzer) equipped with a glass electrode (Orion 91–01) and a single junction reference electrode (Orion 90–02) was used for the potentiometric titrations. Before and after titrations of the sample solutions, the glass electrode was calibrated as a pH probe by titrating known amounts of HNO_3_ with CO_2_-free NaOH solutions and determining the equivalence point by Gran’s method [28], which then determines the standard potential, *E*_0_, and the liquid junction potential, *j*. 40 cm^3^ solutions of 0.002 mol·L^−1^ Na_5_P_3_O_10_ or Na_5_P_3_O_8_(NH)_2_ + 0.10 mol·L^−1^ NaNO_3_ were titrated stepwise by a solution of 0.01 mol·L^−1^ HNO_3_ + 0.10 mol·L^−1^ NaNO_3_. All titrations were carried out at least three times, and all titration results showed good agreement with each other.

### Schwarzenbach’s Titration Procedure for the Determination of the Complex Stability Constants

A Schwarzenbach’s titration procedure was employed for the determination of the stability constants of the ML-type complexes for various divalent metal ions [32–35]. All titrations were carried out at 5.0, 15.0, 25.0, 30.0, or 35.0 (±0.5) °C under a N_2_ atmosphere. Before and after the titrations of the sample solutions, the glass electrode was calibrated by Gran’s method [28]. 20 cm^3^ solutions of 0.002 mol·L^−1^ Na_5_P_3_O_10_ or Na_5_P_3_O_8_(NH)_2_ + 0.002 mol·L^−1^ M(NO_3_)_2_ (M; Mg, Ca, Sr, Ba, Ni, Cu, and Zn) + 0.010 mol·L^−1^ HNO_3_ + 0.10 mol·L^−1^ NaNO_3_ were titrated stepwise by a solution of 0.04 mol·L^−1^ NaOH + 0.10 mol·L^−1^ NaNO_3_. All titrations were carried out at least three times, and all titrations showed good agreement with each other. Other details are the same as for the determination of the stepwise protonation constants.

## Results and Discussion

The first two and three protonation steps of the ${\text{P}}_{ 3} {\text{O}}_{10}^{5 - }$ and ${\text{P}}_{ 3} {\text{O}}_{ 8} ( {\text{NH}})_{2}^{5 - }$ ligands, respectively, were considered in the current work in the pH range 2.5–9 using the Schwarzenbach titration procedure. Protonation equilibria for ${\text{H}}_{m} {\text{P}}_{3} {\text{O}}_{(10 - n)} ({\text{NH}})_{n}^{m - 5}$ (*m* = 0–3, *n* = 0, 2) occur in Na_5_P_3_O_(10–*n*)_(NH)_*n*_ (*n* = 0, 2) aqueous solutions, and the equations of the mass action law for the protonation equilibria are as follows:

1 $$ {\text{HL}}^{{ 4{-}}} \mathop \rightleftharpoons \limits^{{K_{ 1} }} {\text{H}}^{ + } + {\text{ L}}^{{ 5{-}}} \quad \quad \quad K_{1} = \frac{{[{\text{H}}^{ + } ][{\text{L}}^{5 - } ]}}{{[{\text{HL}}^{4 - } ]}} $$

2 $$ {\text{H}}_{ 2} {\text{L}}^{{ 3{-}}} \mathop \rightleftharpoons \limits^{{K_{ 2} }} {\text{H}}^{ + } + {\text{ HL}}^{{ 4{-}}} \quad \quad \quad K_{2} = \frac{{[{\text{H}}^{ + } ][{\text{HL}}^{4 - } ]}}{{[{\text{H}}_{ 2} {\text{L}}^{3 - } ]}} $$

3 $$ {\text{H}}_{ 3} {\text{L}}^{{ 2{-}}} \mathop \rightleftharpoons \limits^{{K_{ 3} }} {\text{H}}^{ + } + {\text{ H}}_{ 2} {\text{L}}^{{ 3{-}}} \quad \quad \quad K{}_{3} = \frac{{[{\text{H}}^{ + } ][{\text{H}}_{ 2} {\text{L}}^{3 - } ]}}{{[{\text{H}}{}_{ 3}{\text{L}}^{2 - } ]}} $$

The average number of bound H^+^ ions per ${\text{H}}_{m} {\text{P}}_{3} {\text{O}}_{(10 - n)} ({\text{NH}})_{n}^{m - 5}$ (*m* = 0–5, *n* = 0, 2), $\overline{n}$, can be calculated by dividing by the concentration of bound H^+^ ions, [H^+^]_b_, and $\overline{n}$ can be determined as follows:

4 $$ \bar{n} = \frac{{ [ {\text{H}}^{ + } ]_{\text{b}} }}{{C_{\text{L}} }} = \frac{{ (C_{\text{H}} - [ {\text{H}}^{ + } ] )}}{{C_{\text{L}} }} = \frac{{K_{ 1} [{\text{H}}^{ + } ] + 2K_{ 1} K_{2} [{\text{H}}^{ + } ]^{2} + 3K_{ 1} K_{2} K_{3} [{\text{H}}^{ + } ]^{3} }}{{ 1+ K_{ 1} [{\text{H}}^{ + } ] + K_{ 1} K_{2} [{\text{H}}^{ + } ]^{2} + K_{ 1} K_{2} K_{3} [{\text{H}}^{ + } ]^{3} }} $$

**Fig. 2:**
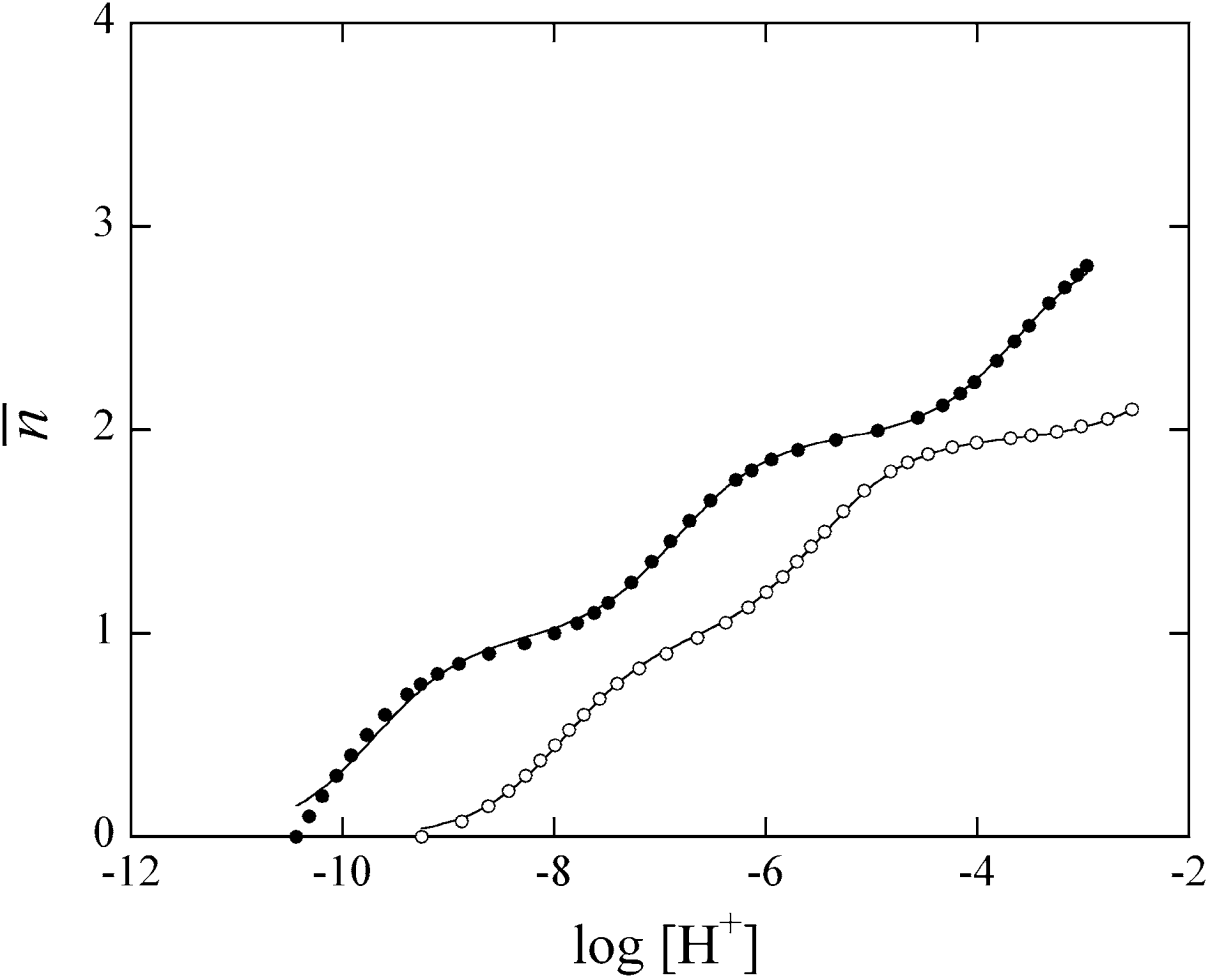
Potentiometric titration curves for the protonation for ${\text{P}}_{ 3} {\text{O}}_{(10 - n)} ({\text{NH}})_{n}^{5 - n}$ (*n* = 0, 2) anions at 25.0 ± 0.5 °C and *I* = 0.1 mol·L^−1^ (NaNO_3_). *Solid lines* refer to the calculated curves by the use of the pertinent parameters of Table 1: (*open circle*), ${\text{H}}_{n} {\text{P}}_{ 3} {\text{O}}_{10}^{n - 5}$ (*n* = 0–2); (*filled circle*), ${\text{H}}_{n} {\text{P}}_{3} {\text{O}}_{8} ({\text{NH}})_{2}^{n - 5}$ (*n* = 0–2)

Figure 2 shows the acid dissociation curves (i.e., plots of $\overline{n}$ versus log_10_ [H^+^]) for the ${\text{P}}_{ 3} {\text{O}}_{10}^{5 - }$ and ${\text{P}}_{ 3} {\text{O}}_{ 8} ( {\text{NH}})_{2}^{5 - }$ anions. The dissociation curve for ${\text{P}}_{ 3} {\text{O}}_{ 8} ( {\text{NH}})_{2}^{5 - }$ is shifted to a higher pH region compared with that for ${\text{P}}_{ 3} {\text{O}}_{10}^{5 - }$. This result indicates that the affinity of the P_3_O_8_(NH)_2_ ligand for protons is greater than that of the P_3_O_10_ ligand [22, 24, 36], because the P_3_O_8_(NH)_2_ ligand contains imino groups [22]. The stepwise protonation constants were determined at all temperatures according to the nonlinear least-squares curve-fitting method using Eq. 4, and the resulting values are listed in Table 1. The protonation constants of the ${\text{P}}_{ 3} {\text{O}}_{10}^{5 - }$ and ${\text{P}}_{ 3} {\text{O}}_{ 8} ( {\text{NH}})_{2}^{5 - }$ anions increase with increasing temperature, indicating that the protonation reactions of these anions are endothermic processes.

**Table 1:** Logarithmic stepwise protonation constants of ${\text{P}}_{ 3} {\text{O}}_{(10 - n)} ({\text{NH}})_{n}^{5 - }$ (*n* = 0, 2) anions determined by potentiometric titrations at *I* = 0.1 mol·L^−1^ (NaNO_3_)

	${\text{P}}_{ 3} {\text{O}}_{10}^{5 - }$		${\text{P}}_{ 3} {\text{O}}_{ 8} ( {\text{NH}})_{2}^{5 - }$		
	log_10_ *K*_1_	log_10_ *K*_2_	log_10_ *K*_1_	log_10_ *K*_2_	log_10_ *K*_3_
5 °C	7.33 (0.01)	5.12 (0.02)	9.26 (0.04)	6.47 (0.04)	3.06 (0.03)
15 °C	7.55 (0.01)	5.22 (0.01)	9.38 (0.04)	6.52 (0.03)	3.13 (0.04)
25 °C	7.69 (0.01)	5.34 (0.01)	9.45 (0.03)	6.65 (0.04)	3.21 (0.04)
30 °C	7.77 (0.02)	5.40 (0.02)	9.50 (0.05)	6.74 (0.02)	3.29 (0.03)
35 °C	7.89 (0.02)	5.49 (0.01)	9.58 (0.04)	6.81 (0.03)	3.35 (0.03)

Numbers in parentheses indicate standard deviations derived from the nonlinear least-squares approximation

In the current study, normal 1:1 complexes were predominantly formed during the titration of 1:1 mixtures of the ligand with a variety of different divalent metal ions, and the resulting equilibria and equation of the mass action law can be described as follows:

5 $$ {\text{M}}^{ 2+ } + {\text{ L}}^{{ 5{-}}} \rightleftharpoons {\text{ML}}^{{ 3{-}}} \quad \quad \quad \beta_{\text{ML}} = \frac{{[{\text{ML}}^{3 - } ]}}{{[{\text{M}}^{2 + } ][{\text{L}}^{5 - } ]}} $$

when the degree of complex formation is sensitive to the pH of the reaction solutions (i.e., the degree of complex formation undergoes measurable changes as the pH changes), potentiometric measurement of the pH of reaction solution can be used to determine the complex formation constants [37]. The metal ion can therefore be displaced from the ligand by a hydrogen ion, which has a comparable affinity for the ligand, and the progress of the displacement can be followed if the complexation equilibrium is sensitive to the pH of the aqueous solution [32, 34]. The stability constants of ML-type complexes of the ${\text{P}}_{ 3} {\text{O}}_{10}^{5 - }$ and ${\text{P}}_{ 3} {\text{O}}_{ 8} ( {\text{NH}})_{2}^{5 - }$ anions with a variety of different divalent metal ions, log_10_ *β*_ML_, were obtained from the experimental data with the aid of the BEST program produced by Martell et al. [38].

**Fig. 3:**
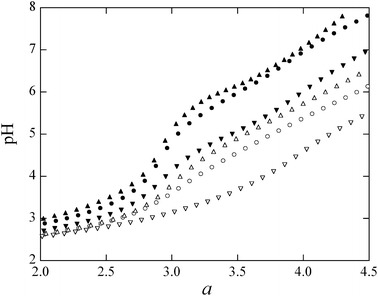
Representative plots of pH versus *a* as Schwarzenbach’s titration curves for divalent metal complexes of ${\text{P}}_{ 3} {\text{O}}_{(10 - n)} ({\text{NH}})_{n}^{5 - }$ (*n* = 0, 2) anions at 25.0 ± 0.5 °C and *I* = 0.1 mol·L^−1^ (NaNO_3_): (*open circle*), Mg^2+^; (*triangle*), Ca^2+^; (*inverted* *triangle*), Cu^2+^. *Open symbols* ${\text{P}}_{ 3} {\text{O}}_{10}^{5 - }$, *filled symbols* ${\text{P}}_{ 3} {\text{O}}_{ 8} ( {\text{NH}})_{2}^{5 - }$. *a* denotes the number of moles of base added per mole of ligand. Details of the titration are given in the text

Figure 3 shows representative titration curves for the P_3_O_10_ and P_3_O_8_(NH)_2_ ligands resulting from the complexation reactions with Mg^2+^, Ca^2+^, and Cu^2+^, evaluated using Schwarzenbach’s procedure. The titration curves for both ligands contain inflection points in the pH range of 3.5–4.5, which indicates formation of a 1:1 complex. These titration curves are clearly shifted to a higher *a* value in the region of *a* > 2.8, where *a* represents the number of moles of alkali added per mole of ligand. The extent to which these shifts occurred in the titration curves indicates that the P_3_O_10_ and P_3_O_8_(NH)_2_ ligands formed complexes of greater stability with the transition metal ion Cu^2+^ than with the alkaline earth metal ions Mg^2+^ and Ca^2+^ [35, 39].

The stability constant (log_10_ *β*_ML_) values determined for the ML-type complexes of the different divalent metal ions of the P_3_O_10_ and P_3_O_8_(NH)_2_ ligands are listed in Table 2. The log_10_ *β*_ML_ values clearly increase as the basicities of the ligands increase, because the basicities of the phosphate groups of the P_3_O_8_(NH)_2_ ligand are greater than those of the free P_3_O_10_ ligand. A critical comparison of our data with other data previously published in the literature by several research groups revealed some interesting differences. For example, differences of as much as tenfold have been reported in the stability constant data in comparison with the values determined in the current study [40, 41]. The stability constants of the Mg–P_3_O_10_ complex determined by Lambert et al. [40] and those of the Zn– and Cu–P_3_O_10_ complexes determined by Andress et al. [41] appear to be ambiguous and inaccurate, because these values were not determined using a non-linear data analysis with a computer program, and do not therefore represent unique values over the entire pH range. This is important, because the values of these constants are dependent upon the pH of the reaction mixture. The reliability of the stability constant of the Cu–P_3_O_10_ (log_10_ *β*_ML_ = 8.70) complex was determined by Sturrock et al. [42], and this value appeared to reflect a greater degree of accuracy in spite of differences in the experimental methods used, in that the potentiometry was conducted with a glass electrode in the current study, whereas polarography was used by Sturrock et al. The differences in the values between the studies in this case appear to originate from differences in the supporting electrolyte and the ionic strength.

**Table 2:** Logarithmic stability constants, log_10_ *β*_ML_, of various divalent metal complexes of ${\text{P}}_{ 3} {\text{O}}_{(10 - n)} ({\text{NH}})_{2}^{5 - }$(*n* = 0, 2) anions determined by Schwarzenbach’s titration procedure at *t* = 25.0 ± 0.5 °C, *I* = 0.1 mol·L^−1^ (NaNO_3_)

	${\text{P}}_{ 3} {\text{O}}_{10}^{5 - }$	${\text{P}}_{ 3} {\text{O}}_{ 8} ( {\text{NH}})_{2}^{5 - }$
Mg^2+^	4.58 (0.14)	5.28 (0.03)
Ni^2+^	6.16 (0.13)	6.61 (0.05)
Cu^2+^	8.01 (0.12)	8.24 (0.04)
Zn^2+^	6.55 (0.10)	7.35 (0.07)
Ca^2+^	4.98 (0.14)	5.79 (0.08)
Sr^2+^	4.45 (0.13)	5.13 (0.05)
Ba^2+^	4.15 (0.16)	4.71 (0.07)

Numbers in parentheses indicate standard deviations derived from replicate experiments

In contrast to these differences, several protonation constants [43] and log_10_ *β*_ML_ values [44] have been reported by Cigala et al. [43] and Högfeldt et al. [44], respectively, for the P_3_O_10_ ligand (i.e., log_10_ *Κ*_1_ = 7.86, log_10_ *Κ*_2_ = 5.56, log_10_ *β*_ML_ = 8.20 (Cu^2+^), 6.83 (Zn^2+^), 4.80 (Ca^2+^), 4.00 (Sr^2+^)) under similar experimental conditions to those used in the current study, including the experimental method, supporting electrolyte and ionic strength. Furthermore, the stability constants of Zn– and Cu–P_3_O_10_ complexes (log_10_ *β*_ML_ values of 6.9 and 7.3, respectively) have been determined by Johansson et al. [45] under similar experimental conditions to those used in the current study. It is noteworthy that the log_10_ *β*_ML_ values determined in the current study show good agreement with those from this portion of literature values, which effectively supports the validity and the accuracy of the calculation values listed in Tables 1 and 2.

With regard to the log_10_ *β*_ML_ value of the Zn^2+^–${\text{P}}_{ 3} {\text{O}}_{10}^{5 - }$ complex, the experimental data (log_10_ *β*_ML_ = 6.55) from the current study is slightly different from that reported in the literature (log_10_ *β*_ML_ = 7.20) [44]. Furthermore, to clarify the dominant formation of the normal 1:1 complexes, solutions containing different initial concentrations of the divalent metal ions [i.e., 0.002 mol·L^−1^ Na_5_P_3_O_10_ or Na_5_P_3_O_8_(NH)_2_ + *X* mol·L^−1^ M(NO_3_)_2_ (M; Ca and Cu) + 0.010 mol·L^−1^ HNO_3_ + 0.10 mol·L^−1^ NaNO_3_ (*X* = 0.001 and 0.004)] were also titrated against a solution of 0.04 mol·L^−1^ NaOH + 0.10 mol L^−1^ NaNO_3_, and the resulting titration curves are shown in Fig. S1. In spite of the differences in the initial concentrations of the divalent metal ions, there are no discernible differences between the titration curves and the log_10_ *β*_ML_ values of the anions calculated with the BEST program. These results therefore effectively confirmed the dominance of normal 1:1 complexes.

**Fig. 4:**
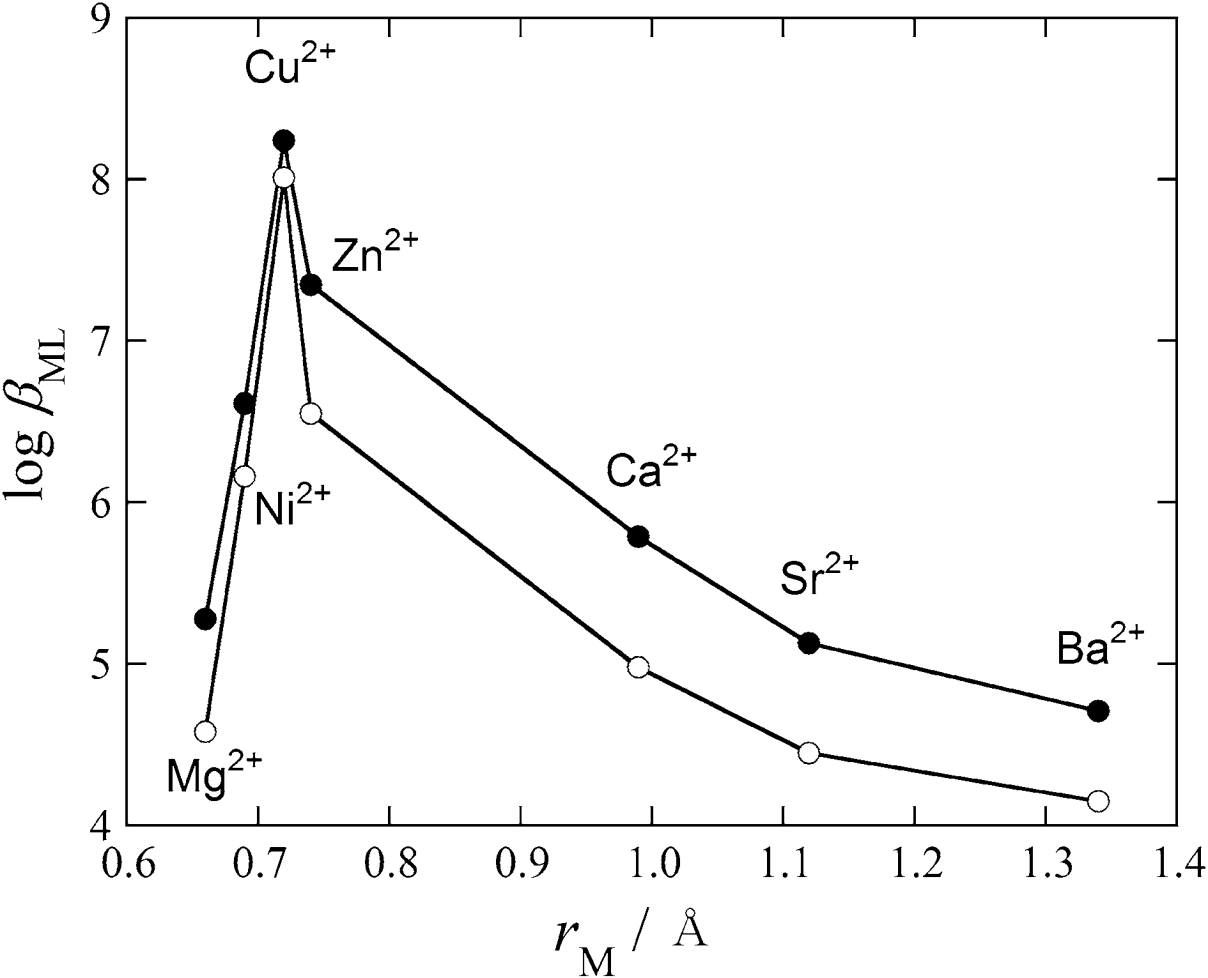
Relationships between log_10_ *β*_ML_ and ionic radii of various divalent metal ions at 25.0 ± 0.5 °C and *I* = 0.1 mol·L^−1^ (NaNO_3_). *Open symbols* P_3_O_10_^5−^, *filled symbols*: ${\text{P}}_{ 3} {\text{O}}_{ 8} ( {\text{NH}})_{2}^{5 - }$

The difference between the log_10_ *Κ*_1_ values of the ${\text{P}}_{ 3} {\text{O}}_{10}^{5 - }$ and ${\text{P}}_{ 3} {\text{O}}_{ 8} ( {\text{NH}})_{2}^{5 - }$ anions is 1.76 (9.45–7.69) at 25 °C on a logarithmic scale, whereas the differences in the log_10_ *β*_ML_ values of the corresponding Cu and Zn complexes are only 0.37 (Cu, 8.38–8.01) and 0.92 (Zn, 7.47–6.55), respectively. It is likely that the specific properties of the metal ions will have a greater effect on the stabilities of the P_3_O_10_ and P_3_O_8_(NH)_2_ complexes with different divalent metal ions than the basicities of the ligands. It has been reported that the log_10_ *β*_ML_ values of *cyclo*-triphosphate and *cyclo*-tri-μ-imidotriphosphate anions can vary significantly following the stereochemical adjustment of the ligand molecule to a metal ion (i.e., the ring size of a ligand molecule and the ionic radius of a metal ion) [21, 46]. Figure 4 shows the dependence of the log_10_ *β*_ML_ values of the P_3_O_10_ and P_3_O_8_(NH)_2_ complexes on the divalent metal ionic radius, *r*_M_. Both the P_3_O_10_ and P_3_O_8_(NH)_2_ ligands show their greatest stabilities in the corresponding Cu complexes, and also clearly show a similar *r*_M_ dependence in terms of their log_10_ *β*_ML_ values, despite the difference in the bridging atoms of the different ligands. Furthermore, the order of the log_10_ *β*_ML_ values for the first-row transition metal ions is in accordance with the Irving–Williams order [47–49]. These results suggest that only the non-bridging oxygen atoms are coordinating directly to the different metal ions, and that the imino groups do not participate in the coordination to the metal ions.

A comparison of the stabilities of the complexes on their own is insufficient when discussing the effects of the different functional groups and bridging atoms of a series of ligands with similar molecular structures on different complex structures. This is because a specific enthalpy–entropy compensation effect is frequently observed between changes in enthalpy, Δ*H*°, and the entropy, Δ*S*°, during the complexation reactions of a series of ligands [50–55]. Consequently, changes in the Gibbs energy, Δ*G*°, which only reflect the stability of the complex, tend to be less significant for the estimation of different complex structures. As a consequence of enthalpy–entropy compensation, a mere comparison of the complex stabilities may not accurately reflect the coordination structures of the complexes of a ligand [50]. Thus, in order to consider the detailed coordination structures of the divalent metal complexes of P_3_O_10_ and P_3_O_8_(NH)_2_ ligands, a van’t Hoff plot (i.e., the temperature dependence of ln *β*_ML_) (Table S1) was employed to evaluate the thermodynamic parameters of the complexation reactions, Δ*H*° and Δ*S*° [56].

**Fig. 5:**
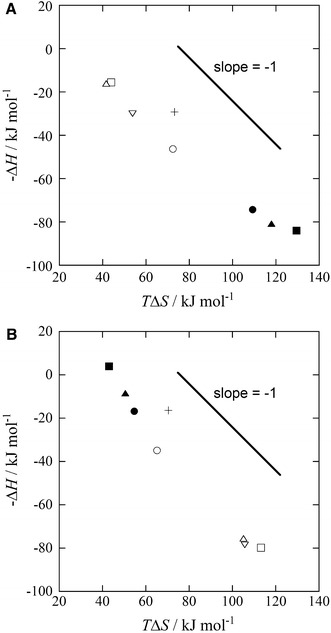
Relationships between −Δ*H*° and *T*Δ*S*° for the complexation of ${\text{P}}_{ 3} {\text{O}}_{(10 - n)} ({\text{NH}})_{n}^{5 - }$ (*n* = 0, 2) anions with various divalent metal ions at 25.0 ± 0.5 °C and *I* = 0.1 mol·L^−1^ (NaNO_3_). **a** ${\text{P}}_{ 3} {\text{O}}_{10}^{5 - }$; **b** ${\text{P}}_{ 3} {\text{O}}_{ 8} ( {\text{NH}})_{2}^{5 - }$. (*plus*), H^+^; (*open circle*), Mg^2+^; (*filled circle*), Ni^2+^; (*filled square*), Cu^2+^; (*filled triangle*), Zn^2+^; (*open square*), Ca^2+^; (*open triangle*), Sr^2+^; (*inverted triangle*), Ba^2+^. *Open symbols* refer to alkaline earth metal ions, and *closed symbols* refer to first row transition metal ions

**Fig. 6:**
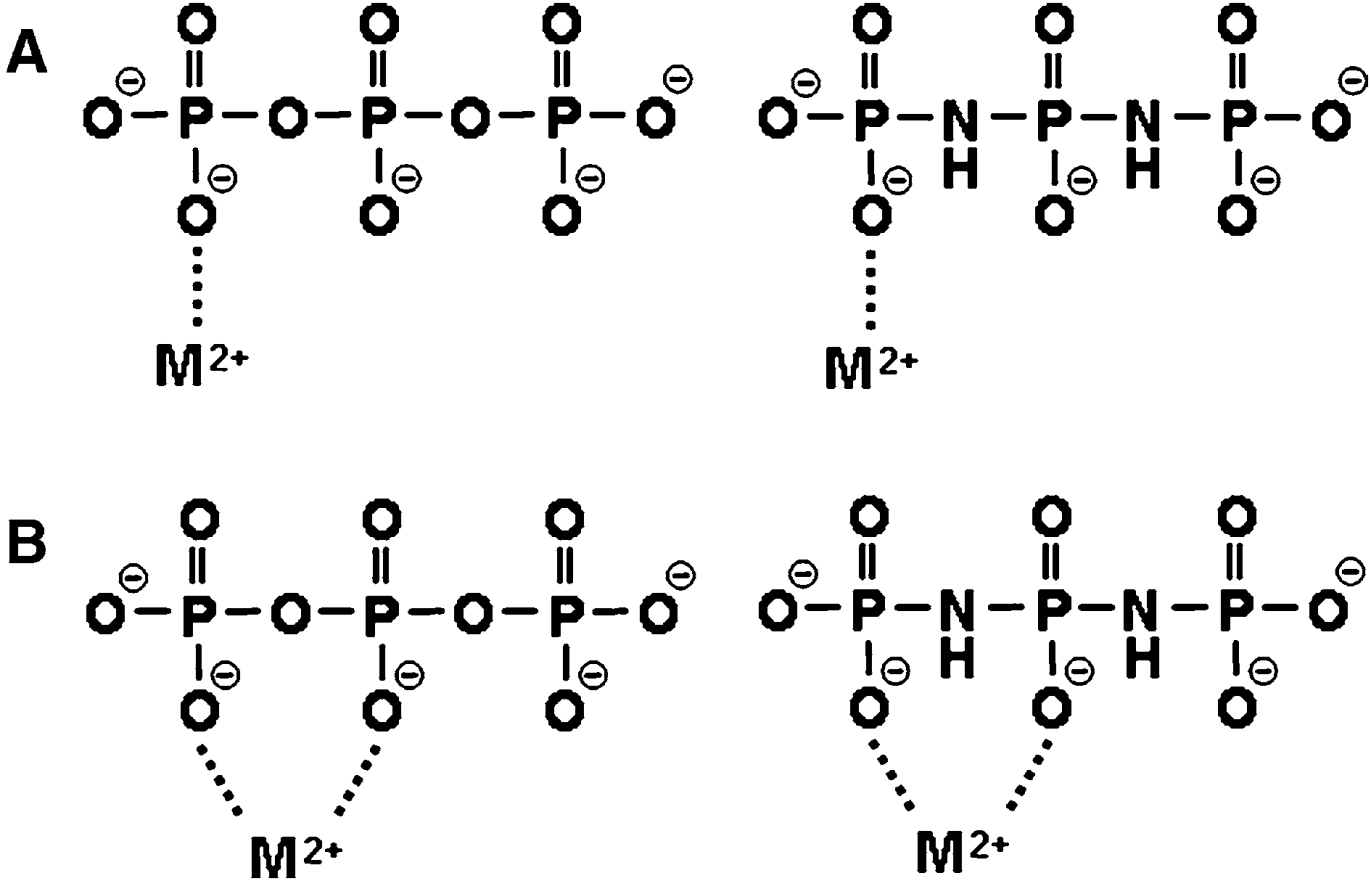
Divalent metal ion binding modes for the complexation of${\text{P}}_{ 3} {\text{O}}_{(10 - n)} ({\text{NH}})_{n}^{5 - }$ (*n* = 0, 2) anions with various divalent metal ions. Structure **a** is considered to be an enthalpically favorable monodentate complex, while structure **b** is considered to be an entropically favorable bidentate complex. There are indications that at equilibrium an inner-sphere binding mode occurs, i.e., the nonbridging oxygen atoms in a ligand and the metal ion are not separated by water molecules

The van’t Hoff plots obtained in the current study give straight lines for all of the complexation systems (Fig. S2). The −Δ*H*° and *T*Δ*S*° values are listed in Table 3, and Fig. 5 shows the relationships between the −Δ*H*° and *T*Δ*S*° values for the complexation reactions of the P_3_O_10_ and P_3_O_8_(NH)_2_ ligands. The slopes of the −Δ*H*° versus *T*Δ*S*° plots have a gradient of *ca.* −1, which indicates a specific enthalpy–entropy compensation effect. In all of the complex formation systems of the P_3_O_10_ and P_3_O_8_(NH)_2_ ligands evaluated in the current study, the complexation reactions of the Ca^2+^, Sr^2+^, Ba^2+^–${\text{P}}_{ 3} {\text{O}}_{10}^{5 - }$ and Cu^2+^, Zn^2+^, Ni^2+^–${\text{P}}_{ 3} {\text{O}}_{ 8} ( {\text{NH}})_{2}^{5 - }$ systems are particularly enthalpically favored, suggesting that each metal ion forms a monodentate complex with a strong coordination to each ligand (Fig. 6a). In contrast, the complexation reactions of the Cu^2+^, Zn^2+^, Ni^2+^–${\text{P}}_{ 3} {\text{O}}_{ 8} ( {\text{NH}})_{2}^{5 - }$ and Ca^2+^, Ba^2+^, Sr^2+^–${\text{P}}_{ 3} {\text{O}}_{ 8} ( {\text{NH}})_{2}^{5 - }$ systems are particularly entropically favored, suggesting the formation of bidentate complexes through the release of several hydration water molecules around the free metal ion to the bulk solution through multidentate coordination, with the ligand forming an entropically stable complex in aqueous solution [48, 56]. In the Cu^2+^, Zn^2+^, Ni^2+^–${\text{P}}_{ 3} {\text{O}}_{10}^{5 - }$ complexation systems, there is no dispute about the coordination structures of the bidentate complexes, with a metal cation coordinating to two non-bridging oxygen atoms in the neighboring phosphate groups, as shown in Fig. 6b.

**Table 3:** Thermodynamic parameters for the complexation reactions of various divalent metal complexes of ${\text{P}}_{ 3} {\text{O}}_{(10 - n)} ({\text{NH}})_{n}^{5 - }$ (*n* = 0, 2) anions *t* = 25.0 °C, *I* = 0.1 mol·L^−1^ (NaNO_3_)

	${\text{P}}_{ 3} {\text{O}}_{10}^{5 - }$			${\text{P}}_{ 3} {\text{O}}_{ 8} ( {\text{NH}})_{2}^{5 - }$		
	−Δ*G*°/kJ·mol^−1^	−Δ*H*°/kJ·mol^−1^	*T*Δ*S*°/kJ·mol^−1^	−Δ*G*°/kJ·mol^−1^	−Δ*H*°/kJ·mol^−1^	*T*Δ*S*°/kJ·mol^−1^
H^+^	44.0 (2.7)	−29.2 (1.4)	73.2 (3.3)	54.0 (1.6)	−16.4 (0.6)	70.4 (2.7)
Mg^2+^	26.1 (3.8)	−46.3 (2.8)	72.4 (4.7)	30.3 (2.8)	−34.9 (2.2)	65.2 (4.0)
Ni^2+^	35.0 (4.7)	−74.3 (3.7)	109.3 (5.0)	37.9 (2.8)	−16.8 (1.1)	54.7 (2.9)
Cu^2+^	45.5 (4.0)	−84.0 (3.1)	129.5 (4.9)	46.9 (2.6)	3.9 (0.2)	43.0 (2.3)
Zn^2+^	37.1 (5.1)	−80.8 (3.6)	117.9 (6.0)	42.0 (2.5)	−8.5 (0.3)	50.5 (3.0)
Ca^2+^	28.3 (2.7)	−15.6 (0.8)	43.9 (4.2)	33.4 (5.2)	−79.8 (4.0)	113.2 (5.9)
Sr^2+^	25.5 (2.2)	−16.1 (0.7)	41.6 (3.9)	29.6 (4.6)	−75.5 (3.3)	105.1 (5.7)
Ba^2+^	23.8 (2.2)	−30.0 (2.1)	53.8 (2.8)	27.2 (4.2)	−78.5 (5.2)	105.7 (5.1)

Numbers in parentheses indicate standard deviations derived from the linear least-squares approximation for van’t Hoff plots

Laurie et al. [57] estimated the coordination structures of the transition-metal complexes of the P_3_O_10_ ligand from the relaxation rates of the ^31^P and ^1^H NMR spectra, which have been corrected for the influence of the paramagnetism of the transition metal ions [57]. Given that a complete correction for the influence of the paramagnetism can be difficult to achieve, there is still some ambiguity surrounding the presumed coordinate structure. In spite of this uncertainty, however, the authors went on to propose the predominant formation of the ML-type bidentate complex at pH < 9 and the formation of the ML_2_ type tetradenate complex at pH > 9. These coordination structures are in good agreement with the structures estimated from the results of the current research in the low pH region, and therefore effectively confirm the validity of the estimated coordination structures used in this study.

Although two possible coordination structures can be postulated for the bidentate complexes of the Ca^2+^, Ba^2+^, and Sr^2+^–${\text{P}}_{ 3} {\text{O}}_{ 8} ( {\text{NH}})_{2}^{5 - }$ systems, only one plausible structure involves the participation of one imino nitrogen atom of the ${\text{P}}_{ 3} {\text{O}}_{ 8} ( {\text{NH}})_{2}^{5 - }$ anion in addition to one non-bridging oxygen atom. This unusual structure was suggested on the basis of the tautomerism that can occur between the phosphate (P–O^−^) and imino (P–NH–P) groups of imidopolyphosphate anions [22, 23]. The tautomerism process may place a negative charge on the nitrogen atom, which would facilitate direct metal ion coordination with the nitrogen atoms. According to the hard and soft acids and bases (HSAB) concept, however, hard metal cations such as Ca^2+^, Ba^2+^ and Sr^2+^ should bind to the harder oxygen atom rather than the softer nitrogen atom of the imidopolyphosphate anion. Consequently, direct coordination with the imino nitrogen atom can now been excluded. It therefore follows naturally that the metal ions must also coordinate to the two non-bridging oxygen atoms in the Ca^2+^, Ba^2+^, Sr^2+^–${\text{P}}_{ 3} {\text{O}}_{ 8} ( {\text{NH}})_{2}^{5 - }$ systems, as shown in Fig. 6b.

For the soft metal cations, including Ni^2+^, Cu^2+^ and Zn^2+^, no remarkable increases in the stability constants of the complexes is suggested, based on the chelate effect or conspicuous entropic predominance in the complex formations; hence, participation of the imino nitrogen atom and the formation of bidentate complexes cannot be considered in the Ni^2+^, Cu^2+^, Zn^2+^–${\text{P}}_{ 3} {\text{O}}_{ 8} ( {\text{NH}})_{2}^{5 - }$ systems. These results imply that the stereochemical adjustment of the ligand molecule to a metal ion may vary significantly as a consequence of slight changes (*ca.* 0.2–0.3 Ǻ) in the distance between the neighboring phosphate groups because of the substitution of bridging oxygen atoms by imino nitrogen atoms, and it can be estimated that the two non-bridging oxygen atoms in the P_3_O_10_ and P_3_O_8_(NH)_2_ ligands coordinate to one metal ion whose ionic radius is suitable for the distance between the neighboring phosphate groups, as shown in Fig. 6b.

## Conclusions

The stepwise protonation constants of ${\text{P}}_{ 3} {\text{O}}_{ 8} ( {\text{NH}})_{2}^{5 - }$ are greater than those of the ${\text{P}}_{ 3} {\text{O}}_{10}^{5 - }$ anion, because the basicity of an imino group is greater than that of a bridging oxygen atom. Following from the determination of the log_10_ *β*_ML_ values using Schwarzenbach’s procedure, a good correlation has been found between the log_10_ *β*_ML_ and log_10_ *K*_1_ values. All of the log_10_ *β*_ML_ values increase as the basicity of the anions increase because of the imino groups in the P_3_O_8_(NH)_2_ ligand. The specific properties of the metal ions, however, probably have a more significant effect on the stabilities of the divalent metal complexes than the basicities of the ligands.

The Cu complexes of the P_3_O_10_ and P_3_O_8_(NH)_2_ ligands show the highest log_10_ *β*_ML_ values, as well as a similar *r*_M_ dependence for the log_10_ *β*_ML_ values. In addition, the order of the log_10_ *β*_ML_ values for first-row transition metal ions of the P_3_O_10_ and P_3_O_8_(NH)_2_ ligands is in accordance with the Irving–Williams order. These observations suggest that only the non-bridging oxygen atoms are coordinating directly to the metal ions for both of these ligands, and that the imino groups can not participate in the coordination to the metal ions.

A specific enthalpy–entropy compensation effect was observed between −Δ*H*° and *T*Δ*S*° for the complexation reactions evaluated in the current study. In addition, in the complexation reactions of the Ca^2+^, Sr^2+^, Ba^2+^–${\text{P}}_{ 3} {\text{O}}_{10}^{5 - }$ and Cu^2+^, Zn^2+^, Ni^2+^–${\text{P}}_{ 3} {\text{O}}_{ 8} ( {\text{NH}})_{2}^{5 - }$ systems, the different metal ions form monodentate complexes having a strong coordination with each ligand (i.e., enthalpically stable complexes). In contrast, in the complexation reactions of the Cu^2+^, Zn^2+^, Ni^2+^–${\text{P}}_{ 3} {\text{O}}_{10}^{5 - }$ and Ca^2+^, Ba^2+^, Sr^2+^–${\text{P}}_{ 3} {\text{O}}_{ 8} ( {\text{NH}})_{2}^{5 - }$ systems, entropically stable complexes are probably formed as a result of the formation of bidentate complexes because of the high stereochemical adjustment between the divalent metal ion and the ligand molecule.

The thermodynamic findings concerning the complexation equilibria between the imidophosphate anion and the different metal ions studied in this work will provide useful information and benchmark values for the complexation properties, coordination properties, and hydrolysis profiles of imidophosphate derivatives such as those used in a variety of inorganic flame retardants.

## Electronic supplementary material

Below is the link to the electronic supplementary material.

Supplementary material 1 (DOCX 177 kb)

Supplementary material 2 (DOCX 177 kb)

Supplementary material 3 (DOCX 949 kb)

## References

[1] Allcock HR (1972). Phosphorus–Nitrogen Compounds.

[2] Allcock HR, Walsh EJ (1972). Phosphonitrilic compounds. XIV. Basic hydrolysis of aryloxy- and spiroarylenedioxycyclophosphazenes. J. Am. Chem. Soc..

[3] Allcock HR (1977). Poly(organophosphazenes)—Unusual new high polymers. Angew. Chem. Int. Ed. Engl..

[4] Gouri ME, Bachiri AE, Hegazi SE, Rafik M, Harfi AE (2009). Thermal degradation of a reactive flame retardant based on cyclotriphosphazene and its blend with DGEBA epoxy resin. Polym. Degrad. Stab..

[5] Liu R, Wang X (2009). Synthesis, characterization, thermal properties and flame retardancy of a novel nonflammable phosphazene-based epoxy resin. Polym. Degrad. Stab..

[6] Fei S, Allcock HR (2010). Methoxyethoxyethoxyphosphazenes as ionic conductive fire retardant additives for lithium battery systems. J. Power Sources.

[7] Gleria M, Bertani R, Jaeger RD, Lora S (2009). Fluorine containing phosphazene polymers. J. Fluor. Chem..

[8] Shin YJ, Ham YR, Kim SH, Lee DH, Kim SB, Park CS, Yoo YM, Kim JG, Kwon SH, Shin JS (2010). Application of cyclophosphazene derivatives as flame retardants for ABS. J. Ind. Eng. Chem..

[9] Lu S, Hamerton I (2002). Recent developments in the chemistry of halogen-free flame retardant polymers. Prog. Polym. Sci..

[10] Çoşut B, Hacıvelioğlu F, Durmuş M, Kılıç A, Yeşilot S (2009). The synthesis, thermal and photophysical properties of phenoxycyclotriphosphazenyl-substituted cyclic and polymeric phosphazenes. Polyhedron.

[11] Kang X, Angell CA (2000). Effect of *N*-substituents on protonation chemistry of trichlorophosphazenes. Inorg. Chim. Acta.

[12] Larsen M, Willett R, Yount RG (1969). Imidodiphosphate and pyrophosphate: possible biological significance of similar structures. Science.

[13] March J (1985). Advanced Organic Chemistry.

[14] Yount RG, Babcock D, Ballantyne W, Ojala D (1971). Adenylyl imidiodiphosphate, an adenosine triphosphate analog containing a P–N–P linkage. Biochemistry.

[15] Tran-Dinh S, Roux M, Ellenberger M (1975). Interaction of Mg^2+^ ions with nucleoside triphosphates by phosphorus magnetic resonance spectroscopy. Nucleic Acids Res..

[16] Smith RM, Martell AE (1976). Critical Stability Constants, Inorganic Complexes.

[17] Oszczapowicz J, Raczyńska E (1984). Amidines. Part 13. Influence of substitution at imino nitrogen atom on p*K*_a_ values of N^1^N^1^-dimethylacetamidines. J. Chem. Soc. Perkin Trans..

[18] Tran-Dinh S, Roux M (1977). A phosphorus-magnetic-resonance study of the interaction of Mg^2+^ with adenyl-5′-yl imidodiphosphate. Binding sites of Mg^2+^ ion on the phosphate chain. Eur. J. Biochem..

[19] Gerlt JA, Demou PC, Mehdi S (1982). Oxygen-17 NMR spectral properties of simple phosphate esters and adenine nucleotides. J. Am. Chem. Soc..

[20] Reynolds MA, Gerlt JA, Demou PC, Oppenheimer NJ, Kenyon GL (1983). Nitrogen-15 and oxygen-17 NMR studies of the proton binding sites in imidodiphosphate, tetraethyl imidodiphosphate, and adenylyl imidodiphosphate. J. Am. Chem. Soc..

[21] Miyajima T, Maki H, Sakurai M, Sato S, Watanabe M (1993). Comparison of the complexation behavior of *cyclo*-imido-triphosphate anions with *cyclo*-triphosphate anions in an aqueous solution. Phosphorus Res. Bull..

[22] Miyajima T, Maki H, Sakurai M, Watanabe M (1995). On the protonation equilibria of *cyclo*-μ-imido-polyphosphate anions(I). Phosphorus Res. Bull..

[23] Miyajima T, Maki H, Sakurai M, Watanabe M (1995). On the protonation equilibria of *cyclo*-μ-imido-polyphosphate anions(II). Phosphorus Res. Bull..

[24] Maki H, Nariai H, Miyajima T (2011). ^9^Be and ^31^P NMR analyses on Be^2+^ complexation with *cyclo*-tri-μ-imido triphosphate anions in aqueous solution. Polyhedron.

[25] Watanabe M, Matsuura M, Yamada T (1981). The mechanism of the hydrolysis of polyphosphates. V. The effect of cations on the hydrolysis of pyro- and triphosphates. Bull. Chem. Soc. Jpn..

[26] Audrith LF (1950). Inorganic Syntheses.

[27] Ootaki, H.: Denki Kagaku **44**, 151–156 (1976)

[28] Gran G (1952). Determination of the equivalence point in potentiometric titrations. Part II. Analyst.

[29] Watanabe M, Hinatase M, Sakurai M, Sato S (1991). The synthesis and thermal property of sodium diimidotriphosphate. Gypsum Limest..

[30] Watanabe M, Sakurai M, Hinatase M, Sato S (1992). Synthesis and thermal property of sodium triimidocyclotriphosphate. J. Mater. Sci..

[31] Conde FL, Prat L (1957). A new reagent for the colorimetric and spectrophotometric determination of phosphorus, arsenic and germanium. Anal. Chim. Acta.

[32] Schwarzenbach G, Ackermann H (1950). Metallkomplexe mit polyaminen I–V. Helv. Chim. Acta.

[33] Schwarzenbach G, Freitag E (1951). Komplexone XIX. Die bildungskonstanten von schwer-metallkomplexen der nitrilo-triessigsäure, komplexone XX. Stabilitätskonstanten von schwermetallkomplexen der äthylendiamin-tetraessigsäure. Helv. Chim. Acta.

[34] Martell AE, Schwarzenbach G (1956). Adenosinphosphate und triphosphat als komplexbildner für Calcium und Magnesium. Helv. Chim. Acta.

[35] Khan MM, Martell AE (1962). Metal chelates of adenosine triphosphate. J. Phys. Chem..

[36] Maki H, Ueda Y, Nariai H (2011). Protonation equilibria and stepwise hydrolysis behavior of a series of thiomonophosphate anions. J. Phys. Chem. B..

[37] Martell AE, Motekaitis RJ (1992). Determination and Use of Stability Constants, 2nd edn., Chap. 3.3.

[38] Martell AE, Motekaitis RJ (1992). Determination and Use of Stability Constants, 2nd edn., Appendix II.

[39] Burai L, Ren J, Kovacs Z, Brücher E, Sherry AD (1998). Synthesis, potentiometry, and NMR studies of two new 1, 7-disubstituted tetraazacyclododecanes and their complexes formed with lanthanide, alkaline earth metal, Mn^2+^ , and Zn^2+^ ions. Inorg. Chem..

[40] Lambert SM, Watters JI (1957). The complexes of magnesium ion with pyrophosphate and triphosphate ions. J. Am. Chem. Soc..

[41] Andress VKR, Nachtrab R (1961). Die bestimmung der komplexbildungskonstanten von Me(II)-triphosphatkomplexen. Z. Anorg. Alleg. Chemie..

[42] Sturrock PE, Loughran ED, Watters JI (1962). A study of the stability and basicity of copper(II) triphosphate complexes using the dropping amalgam electrode. Inorg. Chem..

[43] Cigala RM, Crea F, Stefano CD, Lando G, Manfredi G, Sammartano S (2012). Quantitative study on the interaction of Sn^2+^ and Zn^2+^ with some phosphate ligands, in aqueous solution at different ionic strengths. J. Mol. Liq..

[44] Högfeldt E (1982). Stability Constants of Metal-Ion Complexes Part A: Inorganic Ligands.

[45] Johansson A, Wänninen E (1963). The complexometric analysis of pyro- and triphosphates–I. Talanta.

[46] Maki H, Miyajima T, Ishiguro S, Nariai H, Motooka I, Sakurai M, Watanabe M (1996). ^27^Al NMR study on multidentate complexation behavior of *cyclo*-μ-imido-polyphosphate anions. Phosphorus Res. Bull..

[47] Irving H, Williams RJP (1953). The stability of transition-metal complexes. J. Chem. Soc..

[48] Martell AE, Hancock RD (1996). Metal Complexes in Aqueous Solutions.

[49] Begum ZA, Rahman IMM, Tate Y, Egawa Y, Maki T, Hasegawa H (2012). Formation and stability of binary complexes of divalent ecotoxic ions (Ni, Cu, Zn, Cd, Pb) with biodegradable aminopolycarboxylate chelants (dl-2-(2-carboxymethyl)nitrilotriacetic acid, GLDA, and 3-hydroxy-2,20-iminodisuccinic acid, HIDS) in aqueous solutions. J. Solution Chem..

[50] Ozutsumi K, Ishiguro S (1992). A precise calorimetric study of 18-crown-6 complexes with sodium, potassium, rubidium, caesium, and ammonium ions in aqueous solution. Bull. Chem. Soc. Jpn..

[51] Meo PL, D’Anna F, Gruttadauria M, Riela S, Noto R (2004). Thermodynamics of binding between α- and β-cyclodextrins and some *p*-nitro-aniline derivatives: reconsidering the enthalpy–entropy compensation effect. Tetrahedron.

[52] Inoue Y, Hakushi T (1985). Enthalpy–entropy compensation in complexation of cations with crown ethers and related ligands. J. Chem. Soc. Perkin Trans..

[53] Inoue Y, Hakushi T, Liu Y, Tong LH, Jin DS (1993). Thermodynamics of molecular recognition by cyclodextrins. 1. Calorimetric titration of inclusion complexation of naphthalenesulfonates with alpha-, beta-, and gamma-cyclodextrins: enthalpy–entropy compensation. J. Am. Chem. Soc..

[54] Rekharsky MV, Inoue Y (2000). Chiral recognition thermodynamics of β-cyclodextrin: the thermodynamic origin of enantioselectivity and the enthalpy–entropy compensation effect. J. Am. Chem. Soc..

[55] Linert W, Han LF, Lukovits I (1989). The use of the isokinetic relationship and molecular mechanics to investigate molecular interactions in inclusion complexes of cyclodextrins. Chem. Phys..

[56] Huskens J, Sherry AD (1996). Synthesis and characterization of 1, 4, 7-triazacyclononane derivatives with methylphosphinate and acetate side chains for monitoring free MgII by 31P and 1H NMR spectroscopy. J. Am. Chem. Soc..

[57] Laurie O, Oakes J, Rockliffe JW, Smith EG (1986). Phosphorus and proton nuclear magnetic resonance studies of transition-metal complexes of triphosphate and pyrophosphate in aqueous solution. J. Chem. Soc. Faraday Trans. 1.

