# Supplementary material for: Stabilities of the Divalent Metal Ion Complexes of a Short-Chain Polyphosphate Anion and Its Imino Derivative
Source: J Solution Chem. 2013 Nov 7;42(11):2104–18. doi: 10.1007/s10953-013-0099-2 (PMC3843374; doi:10.1007/s10953-013-0099-2)
Supplement: Supplementary file 1 — Supplementary material 1 (DOCX 177 kb) [file 10953_2013_99_MOESM1_ESM.docx]

**Supplementary Material for:**

**Stabilities of the Divalent Metal Ion Complexes of a Short-chain Polyphosphate Anion and its Imino Derivative**

**Hideshi Maki · Masahiko Tsujito· Makoto Sakurai · Tetsuji Yamada · Hiroyuki Nariai · Minoru Mizuhata**

H. Maki (🖂) · M. Tsujito · T. Yamada · H. Nariai · M. Mizuhata

Department of Chemical Science and Engineering, Graduate School of Engineering, Kobe University, 1-1 Rokkodai-cho, Nada, Kobe, 657-8501, Japan

email: [maki@kobe-u.ac.jp](mailto:maki@kobe-u.ac.jp); telephone/fax: +81 78 803 6187

M. Sakurai

Department of Applied Chemistry, College of Engineering, Chubu University,1200 Matsumoto-cho, Kasugai, Aichi 487-8501, Japan

**Table S1** Logarithmic stability constants, log_10_ *β*_ML_, of various divalent metal complexes of $\text{P}_{3}\text{O}_{(10-n)}(\text{NH})_{n}^{5-}$ (*n* = 0, 2) anions determined by Schwarzenbach’s titration procedure, *I* = 0.1 mol·L^–1^ (NaNO_3_)

(A) *t* = 5.0 ± 0.5 °C

|  | $\text{P}_{3}\text{O}_{10}^{5-}$ | $\text{P}_{3}\text{O}_{8}\text{(NH})_{2}^{5-}$ |
| --- | --- | --- |
| Mg^2+^ | 4.00 (0.03) | 4.90 (0.05) |
| Ni^2+^ | 5.17 (0.05) | 6.42 (0.08) |
| Cu^2+^ | 6.90 (0.04) | 8.25 (0.07) |
| Zn^2+^ | 5.48 (0.04) | 7.26 (0.04) |
| Ca^2+^ | 4.75 (0.12) | 4.85 (0.09) |
| Sr^2+^ | 4.27 (0.05) | 4.20 (0.04) |
| Ba^2+^ | 3.78 (0.06) | 3.75 (0.04) |

(B) *t* = 15.0 ± 0.5 °C

|  | $\text{P}_{3}\text{O}_{10}^{5-}$ | ^^ |
| --- | --- | --- |
| Mg^2+^ | 4.32 (0.06) | 5.08 (0.05) |
| Ni^2+^ | 5.71 (0.06) | 6.56 (0.10) |
| Cu^2+^ | 7.44 (0.04) | 8.25 (0.04) |
| Zn^2+^ | 6.00 (0.08) | 7.33 (0.06) |
| Ca^2+^ | 4.85 (0.03) | 5.37 (0.03) |
| Sr^2+^ | 4.37 (0.05) | 4.79 (0.04) |
| Ba^2+^ | 4.01 (0.11) | 4.34 (0.07) |

(C) *t* = 25.0 ± 0.5 °C

|  | $\text{P}_{3}\text{O}_{10}^{5-}$ | $\text{P}_{3}\text{O}_{8}\text{(NH})_{2}^{5-}$ |
| --- | --- | --- |
| Mg^2+^ | 4.58 (0.09) | 5.28 (0.03) |
| Ni^2+^ | 6.16 (0.08) | 6.61 (0.05) |
| Cu^2+^ | 8.01 (0.07) | 8.24 (0.04) |
| Zn^2+^ | 6.55 (0.05) | 7.35 (0.07) |
| Ca^2+^ | 4.98 (0.09) | 5.79 (0.08) |
| Sr^2+^ | 4.45 (0.08) | 5.13 (0.05) |
| Ba^2+^ | 4.15 (0.11) | 4.71 (0.07) |

(D) *t* = 30.0 ± 0.5 °C

|  | $\text{P}_{3}\text{O}_{10}^{5-}$ | ^^ |
| --- | --- | --- |
| Mg^2+^ | 4.71 (0.09) | 5.39 (0.05) |
| Ni^2+^ | 6.31 (0.07) | 6.68 (0.08) |
| Cu^2+^ | 8.23 (0.06) | 8.21 (0.08) |
| Zn^2+^ | 6.70 (0.09) | 7.37 (0.08) |
| Ca^2+^ | 5.00 (0.05) | 6.08 (0.06) |
| Sr^2+^ | 4.53 (0.07) | 5.43 (0.05) |
| Ba^2+^ | 4.26 (0.06) | 5.02 (0.06) |

(E) *t* = 35.0 ± 0.5 °C

|  | $\text{P}_{3}\text{O}_{10}^{5-}$ | ^^ |
| --- | --- | --- |
| Mg^2+^ | 4.86 (0.04) | 5.56 (0.05) |
| Ni^2+^ | 6.55 (0.05) | 6.75 (0.04) |
| Cu^2+^ | 8.40 (0.07) | 8.17 (0.06) |
| Zn^2+^ | 6.96 (0.11) | 7.44 (0.10) |
| Ca^2+^ | 5.02 (0.10) | 6.33 (0.07) |
| Sr^2+^ | 4.56 (0.07) | 5.60 (0.05) |
| Ba^2+^ | 4.34 (0.08) | 5.20 (0.08) |

Numbers in parentheses indicate standard deviations derived from replicate experiments
