# Supplementary material for: Stabilities of the Divalent Metal Ion Complexes of a Short-Chain Polyphosphate Anion and Its Imino Derivative
Source: J Solution Chem. 2013 Nov 7;42(11):2104–18. doi: 10.1007/s10953-013-0099-2 (PMC3843374; doi:10.1007/s10953-013-0099-2)
Supplement: Supplementary file 2 — Supplementary material 2 (DOCX 177 kb) [file 10953_2013_99_MOESM2_ESM.docx]

(A); Ca^2+^


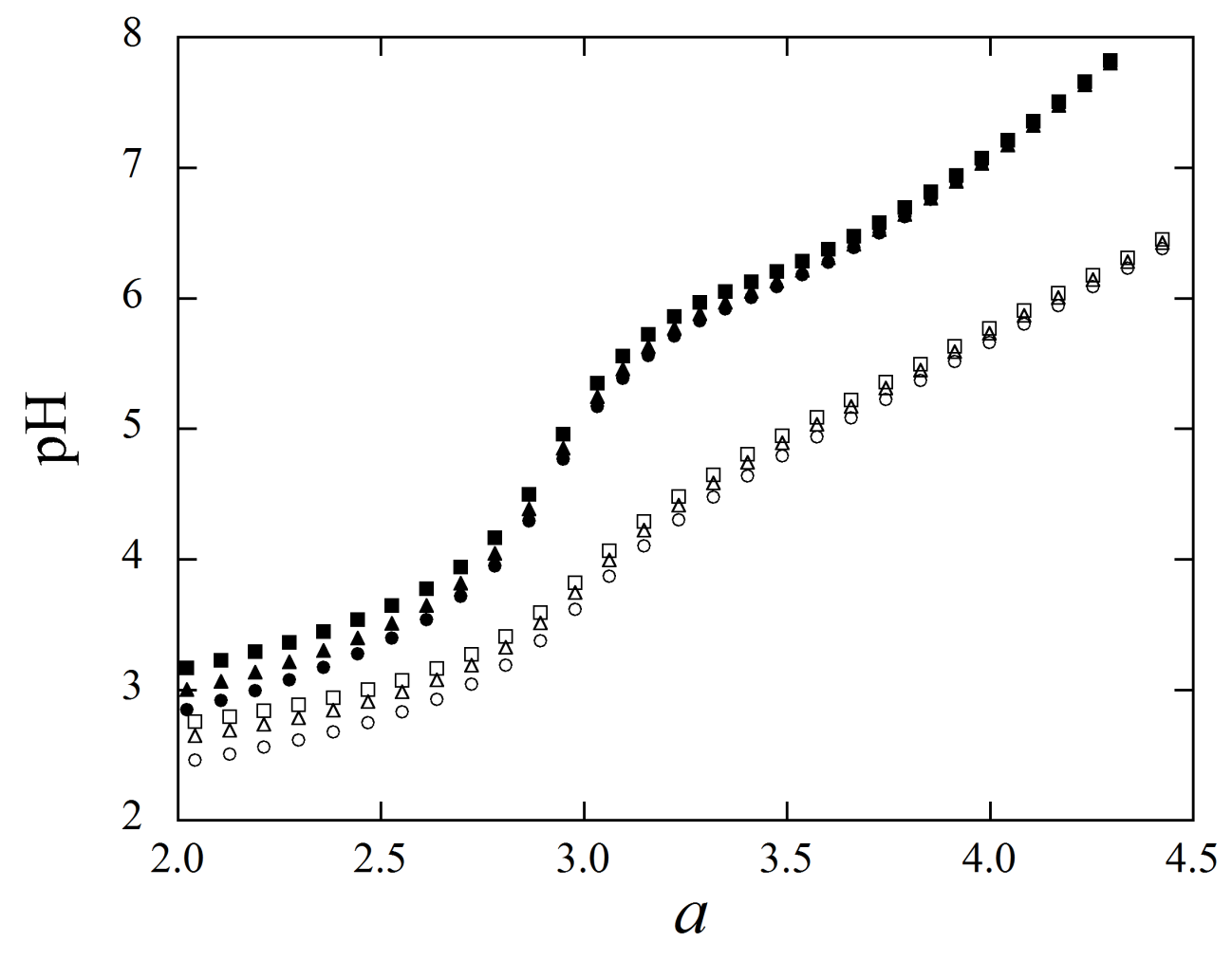


|  | log *β*_ML_ | |
| --- | --- | --- |
| *C*_M_ (mol·L^–1^) | P_3_O_10_^5–^ | P_3_O_8_(NH)_2_^5–^ |
| 0.001 | 4.94 (0.06) | 5.77 (0.07) |
| 0.002 | 4.98 (0.09) | 5.79 (0.08) |
| 0.004 | 5.03 (0.07) | 5.83 (0.08) |

|  | log *β*_ML_ | |
| --- | --- | --- |
| *C*_M_ (mol·L^–1^) | P_3_O_10_^5–^ | P_3_O_8_(NH)_2_^5–^ |
| 0.001 | 7.99 (0.06) | 8.23 (0.05) |
| 0.002 | 8.01 (0.07) | 8.24 (0.04) |
| 0.004 | 8.04 (0.07) | 8.27 (0.04) |


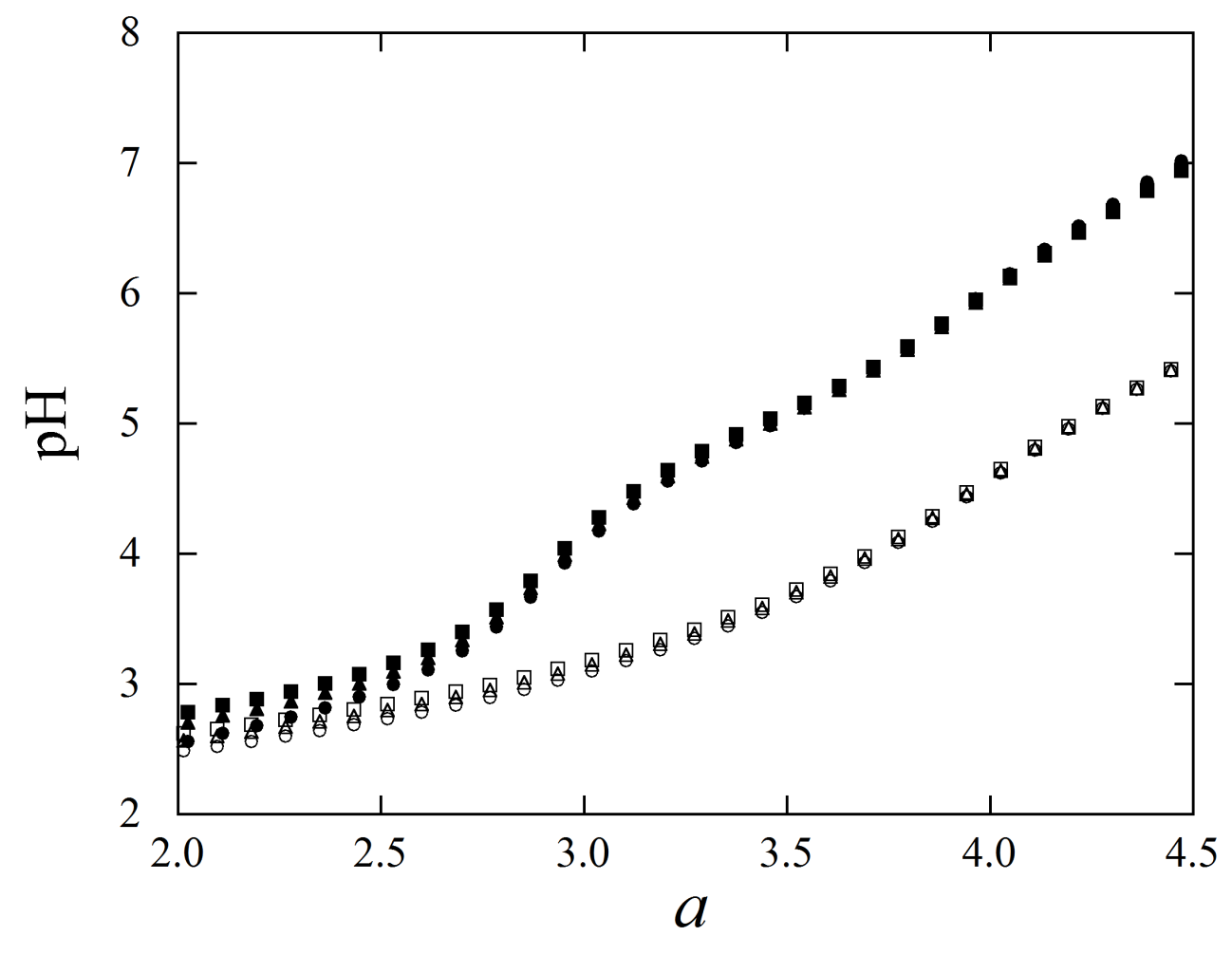


(B); Cu^2+^

Fig. S1 Supplementary Schwarzenbach’s titration curves for the solutions which are different initial concentrations, *C*_M_, of Ca^2+^ or Cu^2+^ ions.

(○) *C*_M_ = 0.001 mol·L^–1^; (△) *C*_M_ = 0.002 mol·L^–1^; (□) *C*_M_ = 0.004 mol·L^–1^. Open: P_3_O_10_^5–^, closed: P_3_O_8_(NH)_2_^5–^. *a* = moles of base added per mole of ligand. The details of the titration are given in the text.
