# Supplementary material for: Stabilities of the Divalent Metal Ion Complexes of a Short-Chain Polyphosphate Anion and Its Imino Derivative
Source: J Solution Chem. 2013 Nov 7;42(11):2104–18. doi: 10.1007/s10953-013-0099-2 (PMC3843374; doi:10.1007/s10953-013-0099-2)
Supplement: Supplementary file 3 — Supplementary material 3 (DOCX 949 kb) [file 10953_2013_99_MOESM3_ESM.docx]

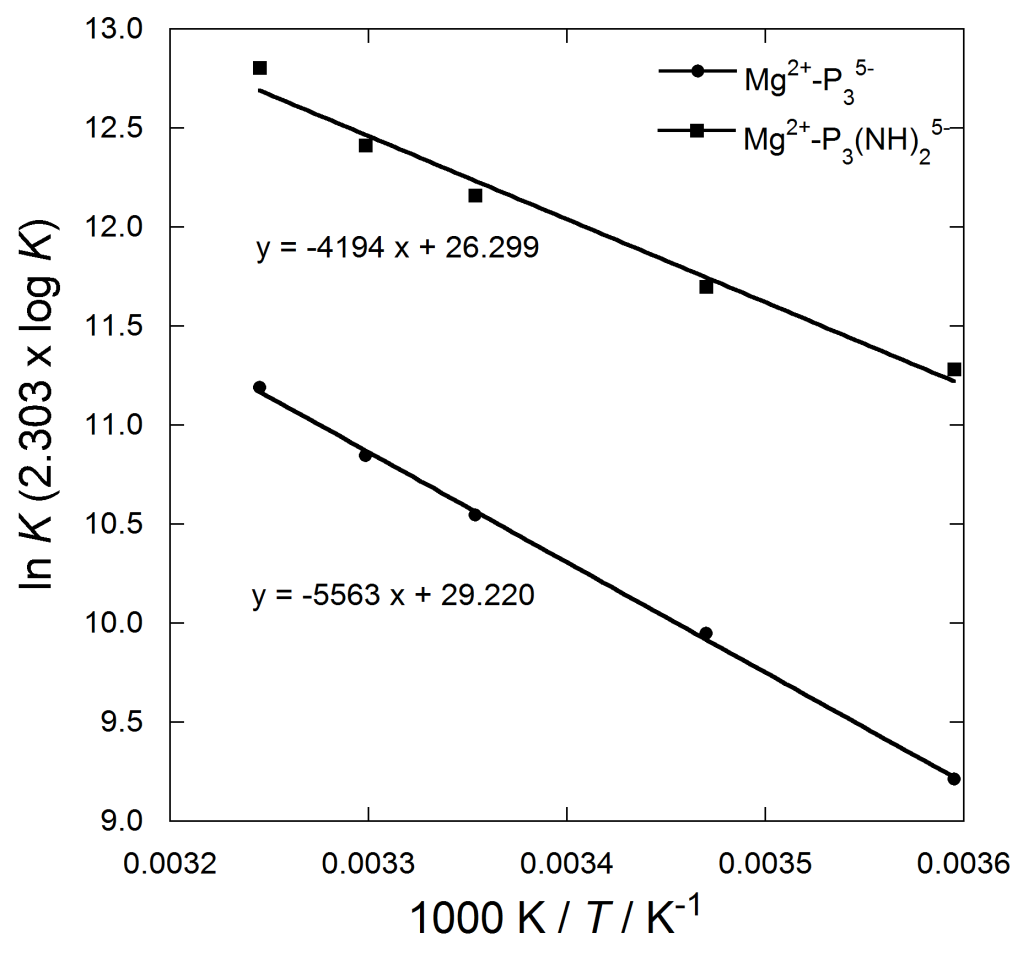

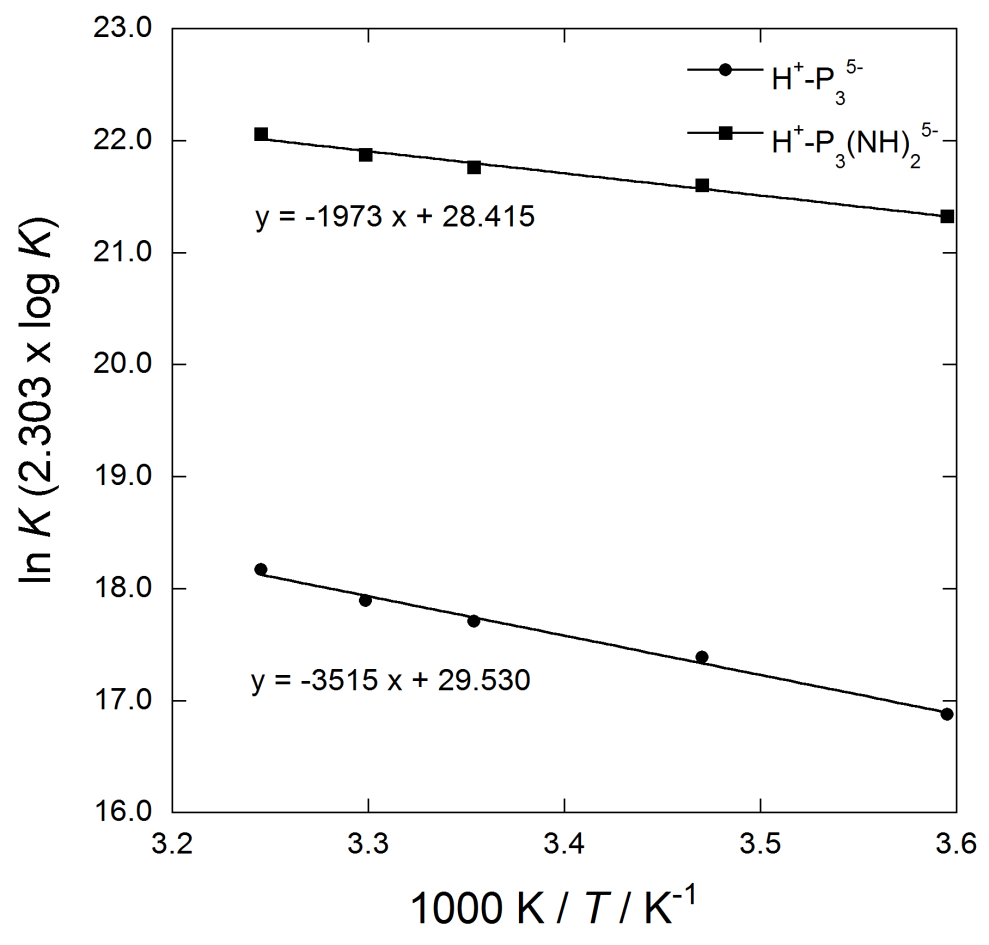


(B)

(A)


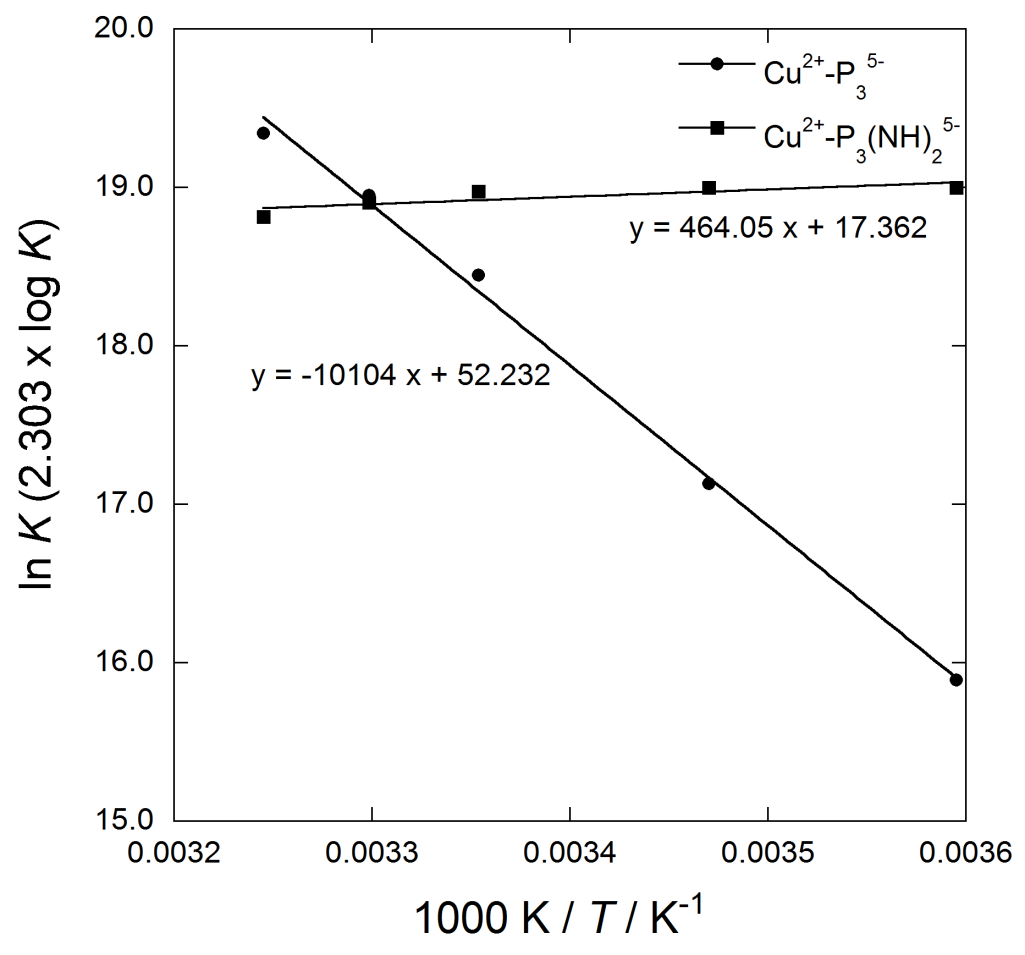

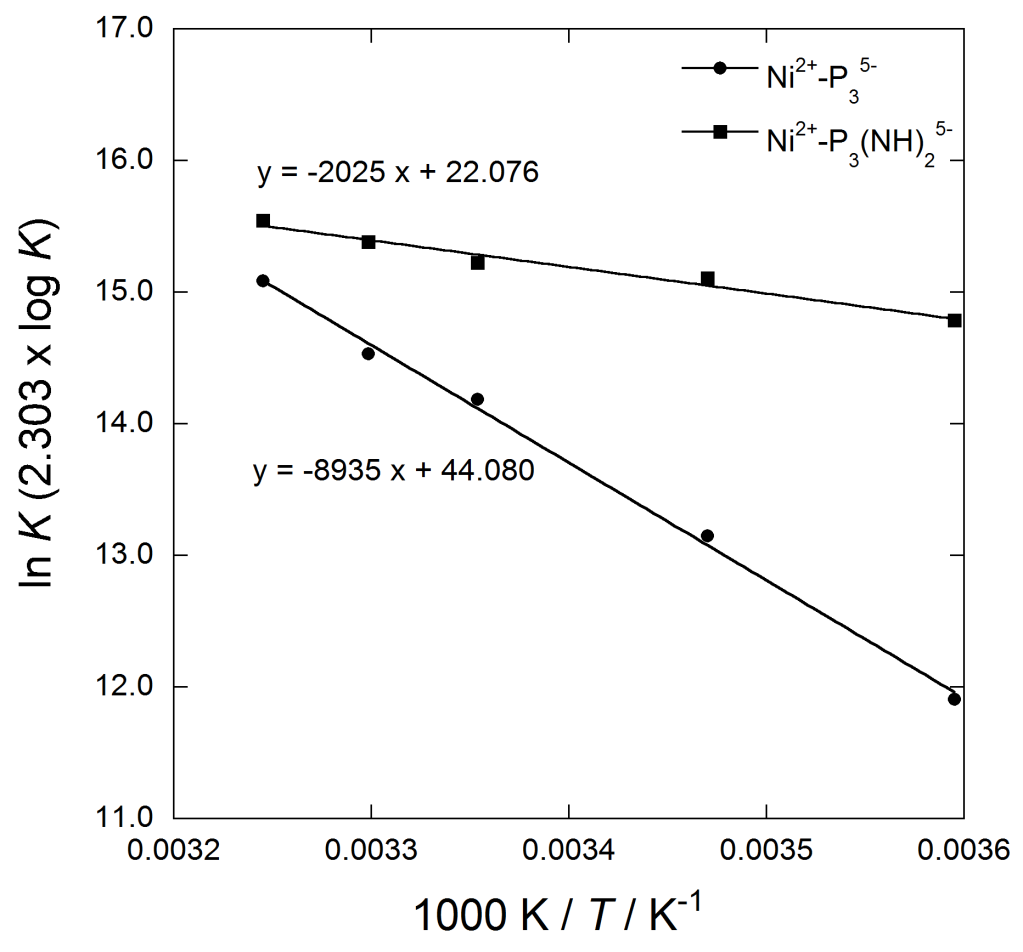


(D)

(C)


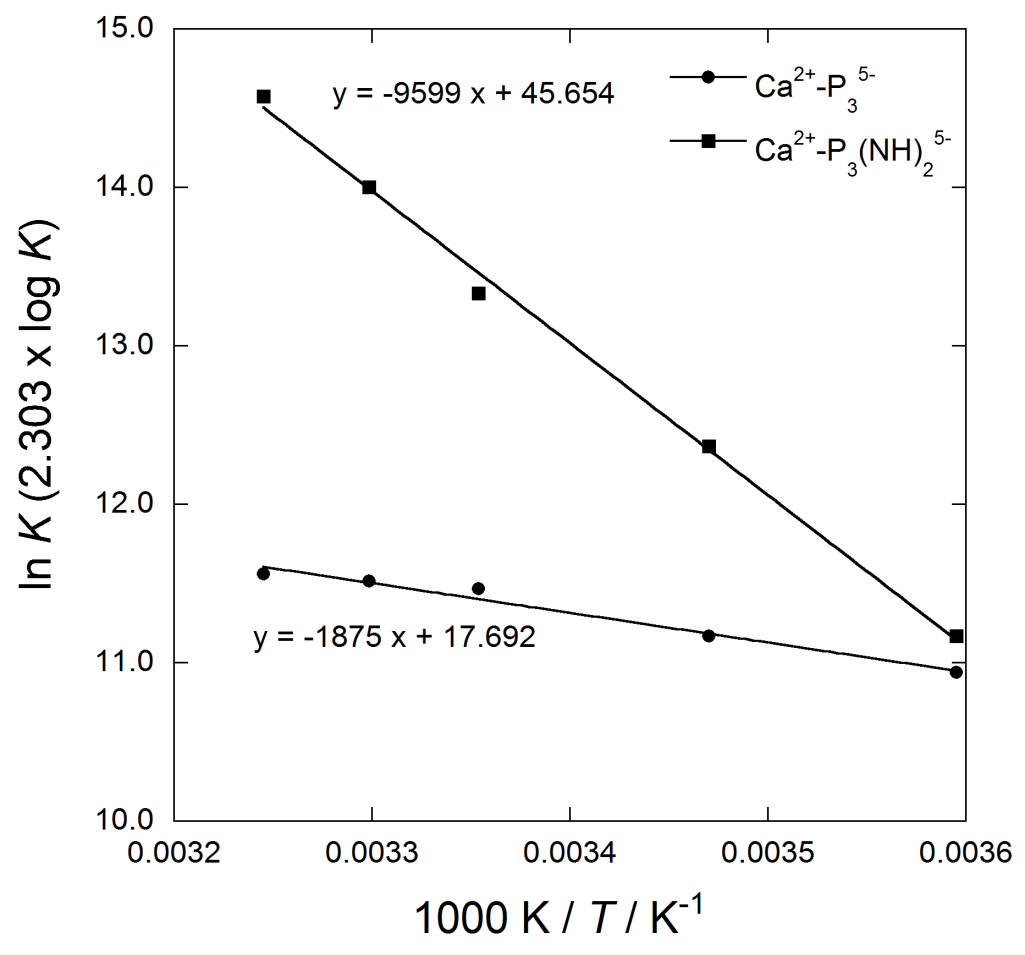

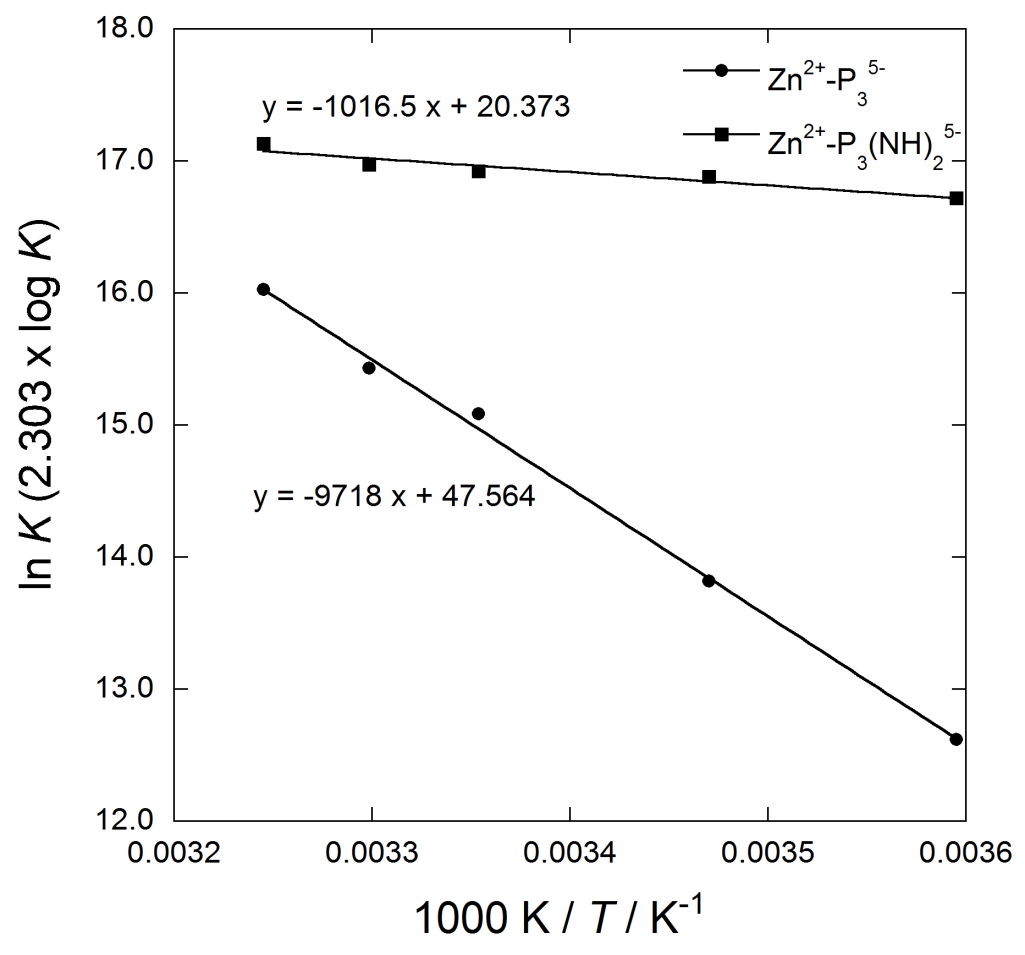


(F)

(E)

(G)

(H)


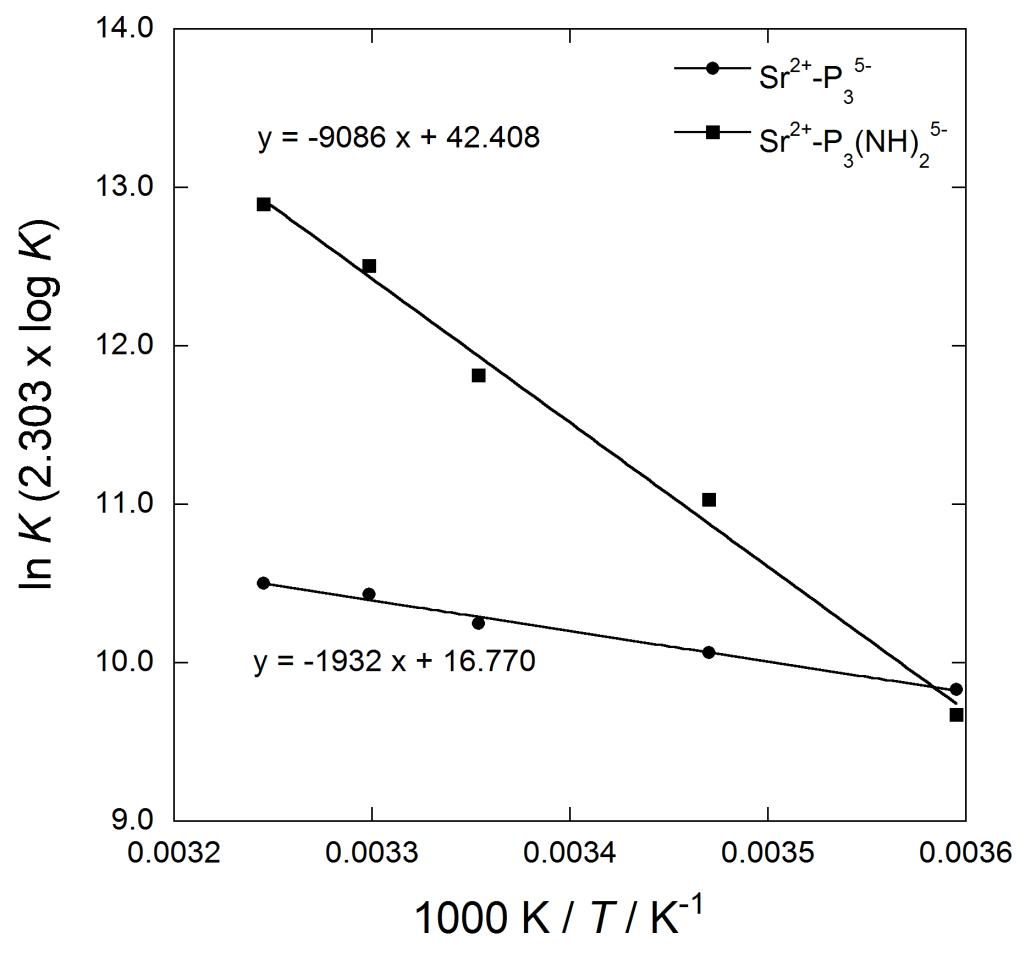


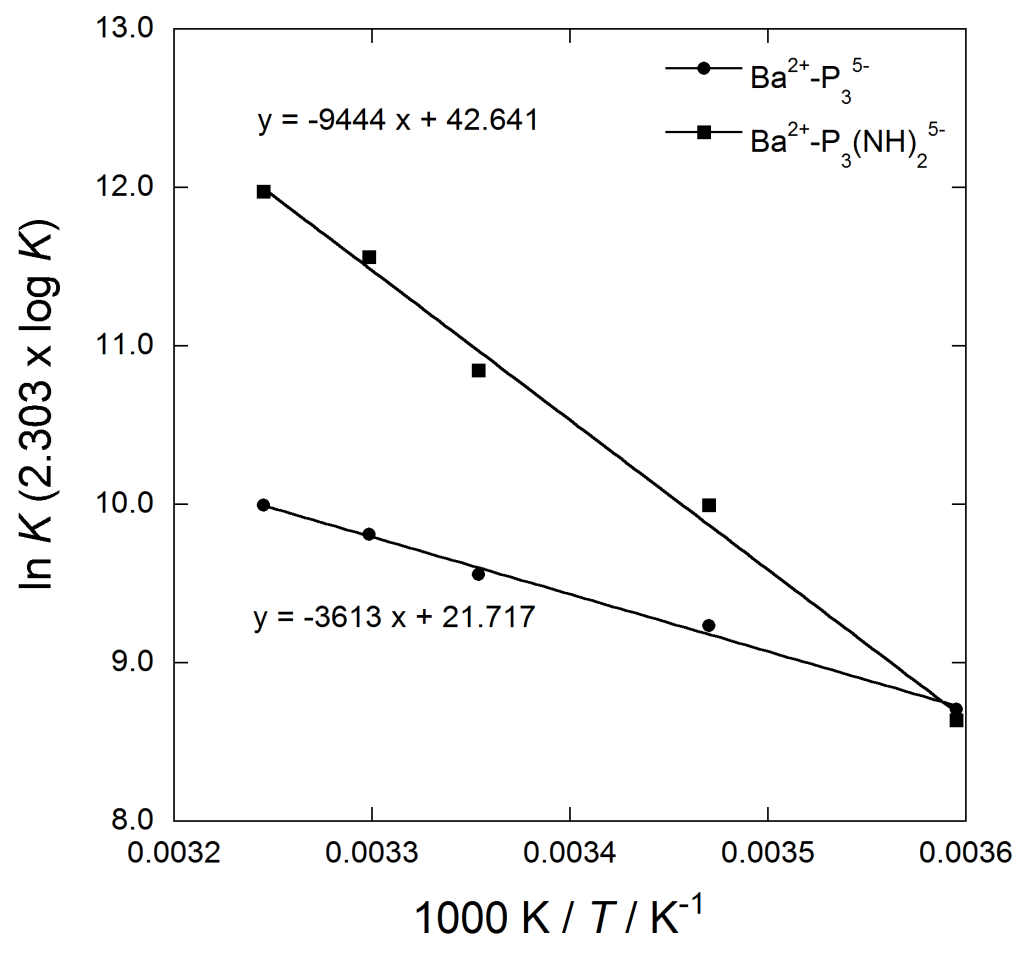


Fig. S2 ln K plotted against reciprocal absolute temperature, *i.e.* van't Hoff plot, for the complex formation reactions of P_3_O_(10–_*_n_*_)_(NH)*_n_*^5–^(*n* = 0, 2) anions. The standard enthalpy changes, Δ*H*°, and the standard entropy changes, *T*Δ*S*°, for the reactions were calculated from the slopes and the intercepts of the straight lines.
